# DNA barcoding reveals a species group of the genus *Campiglossa* (Diptera, Tephritidae, Tephritinae) with recognition of a new species from East Asia and previously unknown females of *Campiglossa coei* (Hardy)

**DOI:** 10.3897/zookeys.899.46779

**Published:** 2019-12-12

**Authors:** Ho-Yeon Han, Kyung-Eui Ro

**Affiliations:** 1 Division of Biological Science and Technology, College of Science and Technology, Yonsei University, 1 Yonseidae-gil, Wonju-si, Gangwon-do 26493, South Korea Yonsei University Wonju-si South Korea

**Keywords:** *
Campiglossa
*, *
Dioxyna
*, *
Homoeotricha
*, *
misella* group, Tephritini

## Abstract

While analyzing DNA barcodes of all the Korean and some East Asian tephritid species in conjunction with the barcode sequences available from BOLD Systems (www.boldsystems.org), the large and taxonomically enigmatic genus *Campiglossa* was recovered as a monophyletic clade, together with the genera *Dioxyna* and *Homoeotricha*, which are here synonymized for that reason. Ten major lineages are also recognized within the *Campiglossa* clade: *producta* group, *loewiana* group, *sororcula* group, *irrorata* group, *achyrophori* group, *difficilis* group, *luxorientis* group, *magniceps* group, *arisanica* group, and *misella* group. Here, more detailed taxonomic accounts are provided for the *misella* group, including four DNA analysis-recovered members: *C. coei*, *C. misella*, *C. paramelaena***sp. nov.**, and *C. melaena*. A single morphological synapomorphy is proposed for this species group: the presence of a large mid-anterior dark wing marking in males with associated structural modification (more apically positioned crossvein R-M than in females). Based on the morphological characteristics, two presumptive members that are only known from male specimens are further recognized: *C. pishanica* and *C. propria* from China. A full description of *C. paramelaena***sp. nov.**, and a redescription of *C. coei*, for which only males were previously known, are provided. For all the included species, a taxonomic key, diagnoses, and photographs to aid their accurate identification are given. Finally, *C. favillacea* is synonymized with *C. coei* and *C. roscida* with *C. misella*, and *C. coei* and *C. pishanica* resurrected from the synonymy of *C. misella*.

## Introduction

Tephritidae is a relatively recently diverged fly family that might have arisen around the Late Eocene (~36 mya; Han and Ro 2016). Currently, this family includes approximately 4,700 valid species under ca. 500 genera, seven of which are species-rich (i.e., over 100 species) genera (Norrbom et al. 1999; Catalogue of Life as of Aug. 2019 – http://www.catalogueoflife.org). These highly diverged genera are notorious for harboring a number of species complexes that are taxonomically difficult to deal with (White 2006; Drew and Romig 2013).

The genus *Campiglossa* Rondani, 1870, is one of those species-rich genera, and is estimated to have approximately 200 described species (White 1988; Norrbom et al. 1999; Catalogue of Life as of Aug. 2019). *Campiglossa* is a predominantly Palaearctic genus but a significant number of representative species occur in all the other zoogeographical regions. The majority of species of known biology are associated with the capitula of composite plants (family Asteraceae) (White 1988). The members of this genus have often been treated either as *Campiglossa* or *Paroxyna* in the past, but Norrbom et al. (1999) did not find any clear distinction between these two genera and, thus, regarded them as a single genus, *Campiglossa*. In the present study, we also synonymize the genera *Dioxyna* Frey, 1945, and *Homoeotricha* Hering, 1944, with the genus *Campiglossa*.

Due to their high intra-specific variation, low inter-specific variation, sexual dimorphism and seasonal variation, systematic investigation of *Campiglossa* is considered very difficult (A. Freidberg, V. Korneyev, S. Masahiro, B. Merz, pers. comm.). Examination of their male and female postabdominal structure has been somewhat helpful for defining species and species groups (White 1988; Korneyev 1990, 1997; Merz 1994; Korneyev and Ovchinnikova 2004). Obtaining host associated specimens has also been useful for understanding their intra- and interspecific variation (Merz 1994; Han 2019). Most recently, DNA barcoding has proven useful for identifying tephritid species and species groups, as well as confirming generic limits (Smit et al. 2013, Barr et al. 2018).

In the process of analyzing DNA barcodes of all the Korean and some East Asian tephritid species in conjunction with the barcode sequences available from BOLD Systems (www.boldsystems.org), we recovered the genus *Campiglossa* as a monophyletic clade together with the genera *Dioxyna* and *Homoeotricha*. We also recognized ten major lineages within the *Campiglossa* clade, each of which can be regarded as a monophyletic species group. In this study, we provide more detailed taxonomic accounts for the *misella* group, including four DNA analysis-recovered members: *C. coei* (Hardy, 1964), *C. misella* (Loew, 1869), *C. paramelaena* sp. nov., and *C. melaena* (Hering, 1941). Based on the morphological characteristics, we further recognize two presumptive members that are only known from male specimens: *C. pishanica* (Wang, 1996) and *C. propria* (Chen, 1938) from China. We provide a full description of *C. paramelaena* sp. nov., and a redescription of *C. coei*, for which only males were previously known. For all included species, we provide a taxonomic key, diagnoses, and photographs to aid their accurate identification.

## Materials and methods

The terminology and morphological interpretations used in this study follow the glossary of White et al. (1999). A total of 12 ratios are used in the descriptions: head ratio (head length excluding the antennae in lateral view/head height); frons-head ratio (narrowest width of frons in dorsal view/width of head); eye ratio (shortest eye diameter/longest eye diameter); gena-eye ratio (genal height/longest eye diameter) - genal height is the distance between ventral eye margin and ventral genal margin anterior to genal seta (gena measured with head tilted slightly dorsally so that gena is at its broadest); antenna-head ratio (antenna length measured from scape to flagellomere 1/head height); arista-antenna ratio (arista length/antenna length); wing-thorax ratio (wing length from tegula to apex of vein R_4+5_/thorax length in dorsal view); wing ratio (wing length/wing width); vein M ratio (distance along vein M between crossveins R-M and DM-Cu/distance between crossveins R-M and BM-Cu); subcosta-costa ratio (distance along vein C of subcostal cell/costal cell); cell r_1_-r_2+3_ ratio (distance along vein C of cell r_1_/cell r_2+3_); cell r_4+5_-r_2+3_ ratio (distance along vein C of cell r_4+5_/cell r_2+3_).

The molecular methods follow Han and Ro (2016, 2018). For our analysis, 765 base pair fragments of the mitochondrial COI gene sequences (the DNA barcode region) were newly obtained from 55 specimens representing 26 species of the genus *Campiglossa*. The collection and voucher data, and GenBank accession numbers (MN445522–MN445576) are presented in Table 1. We used the same PCR and sequencing primers listed in Han and Ro (2016). We analyzed these sequences plus a number of the sequences downloaded from BOLD Systems (www.boldsystems.org, as of Jan. 2019). A neighbor-joining (NJ) analysis (Saitou and Nei 1987) was performed in MEGA version X (Kumar et al. 2018) using the Kimura 2-parameter model of nucleotide substitution (Kimura 1980). A maximum-likelihood (ML) analysis was also performed in MEGA X using the general time reversible model (Nei and Kumar 2000). The reliability of clustering patterns in the ML tree was determined by the bootstrap test (Felsenstein 1985; 2,000 replications). Bayesian inferences (BI) were conducted using MrBayes v3.0b4 (Huelsenbeck and Ronquist 2001) by Markov chain Monte Carlo (MCMC) sampling for two million generations, with tree sampling every 100 generations and a burn-in of 1,000 trees. The BI analyses were run twice using different random starting trees to evaluate the congruence of the likelihood values and posterior clade probabilities (Huelsenbeck et al. 2002). Additional details of the molecular analyses are mentioned in the appropriate section.

**Table 1.** The collection and voucher information for the *Campiglossa* flies sequenced for the DNA barcoding analysis. The status of the voucher specimens and the GenBank accession numbers are indicated in parentheses.

*C. absinthii* (Fabricius, 1805)	1. ♂, KOREA: Gangwon-do, Jeongseon-gun, Nam-myeon, Mt. Mindungsan, from Yupyeong-ri to 1,119 m peak, 37°16'15"N, 128°46'30"E, 4.VIII.2005, H.-Y. Han et al. (both wings glued on a rectangular card; YSUW090915027; GenBank Acc. Nr. MN445522).
	2. ♀, KOREA: Gangwon-do, Jeongseon-gun, Nam-myeon, Mt. Mindungsan, from Yupyeong-ri to 1,119 m peak, 37°16'15"N, 128°46'30"E, 24.VII.2005, H.-Y. Han et al. (both wings glued on a rectangular card; YSUW090915028; GenBank Acc. Nr. MN445523).
	3. ♀, RUSSIA: Primorsky-Krai, Khasansky-District, Barabash, 43°10'46.9"N, 131°28'20.0"E, 22.VI.2008, H.Y. Han & H.S. Lee (specimen with the abdomen detached; YSUW140201102; GenBank Acc. Nr. MN445524).
*C. achyrophori* (Loew, 1869)	1. ♀, SWITZERLAND: Valais 1787–2041 m, Pointe de Bellevue, Morgins, 28.VII.2004, H.-Y. Han & K.-E. Ro (specimen with the abdomen detached; YSUW140201037; GenBank Acc. Nr. MN445525).
*C. albiceps* (Loew, 1873)	1. ♂, USA: North Carolina, Haywood Co, Great Smoky Mountains National Park, in meadow 250 m N of house at Purchase Knob, 1444 m (both wings glued on a rectangular card; YSUW090915005; GenBank Acc. Nr. MN445526).
*C. bidentis* (Robineau-Desvoidy, 1830), comb. nov. from *Dioxyna*	2. ♂, KOREA: Gangwondo, Jeongseon, Nammyeon, Mt. Mindungsan, from Yupyeongri to 1,119 m peak, 37°16'15"N, 128°46'30"E, 16.VII.2005, H.-Y. Han et al. (specimen with the abdomen detached; YSUW130901095; GenBank Acc. Nr. MN445527).
	3. ♀, KOREA: Gyeongsangbuk-do, Bonghwa Myeongho-myeon, Mt. Cheongnyangsan, 29.IX.2007, Coll. H.-S. Lee et al., ex *Bidens biternata* (Lour.) flower, em. 3–12.X.2007 (specimen with the abdomen detached; YSUW130901096; GenBank Acc. Nr. MN445528).
*C. coei* (Hardy, 1964)	1. ♂, CHINA: Yunnan, Mengsong, Manlvcunhanzudazhai, small hilltop, 22°07'44.0"N, 100°28'51.7"E, 1690 m, 12.VII.2011, H.-Y. Han & S.-W. Suk (specimen with the abdomen detached; YSUW 130901058; GenBank Acc. Nr. MN445530).
	2. ♀, CHINA: Yunnan, Mengsong, Bengangxizhai, in forest, 22°10'34.5"N, 100°35'06.8"E, 1725 m, 11.VII.2011, H.-Y. Han & S.-W. Suk (specimen with the abdomen detached; YSUW 130901059; GenBank Acc. Nr. MN445531).
	3. ♂, CHINA: Yunnan, Mengsong, Manlvcunhanzudazhai, small hilltop, 22°07'44.0"N, 100°28'51.7"E, 1690 m, 12.VII.2011, H.-Y. Han & S.-W. Suk (specimen with the abdomen detached; YSUW YSUW140201034; GenBank Acc. Nr. MN445532).
	4. ♀, CHINA: Yunnan, Mengsong, Manlvcunhanzudazhai, small hilltop, 22°07'44.0"N, 100°28'51.7"E, 1690 m, 12.VII.2011, H.-Y. Han & S.-W. Suk (specimen with the abdomen detached; YSUW 140201035 6; GenBank Acc. Nr. MN445533).
*C. deserta* (Hering, 1939)	1. ♂, KOREA: Gangwon-do, Pyeongchang-gun, Doam-myeon, Hoenggye-ri, Daegwallyeong Samyang pasture, col. 7.X.2004, em. 1–21.VI.2005, ex *Aster* sp., flower, H.-Y. Han & H.-W. Byun (both wings glued on a rectangular card; YSUW090915029; GenBank Acc. Nr. MN445534).
	2. ♀, KOREA: Gangwon-do, Jeongseon-gun, Gohan-eup, Mt. Hambaeksan, Recreation forest to Manhang-jae, col. 10.X.2003, em. 24–31.V.2004, ex *Aster ciliosus* Kitamura ?, flower, H.-Y. Han & K.-E. Ro (both wings glued on a rectangular card; YSUW090915030; GenBank Acc. Nr. MN445535).
	3. ♀, RUSSIA: Primorsky-Krai, Nadezhdinsky-District, Vol’no-Nadezhdinskoye, 43°22'31.6"N, 132°01'43.1"E, 22.VI.2008, Coll. H.-Y. Han & H.-S. Lee (specimen with the abdomen detached; YSUW140201103; GenBank Acc. Nr. MN445536).
	1. ♀, KOREA: Gangwon-do, Jeongseon-gun, Nam-myeon, Mt. Mindungsan, from Yupyeong-ri to 1,119 m peak, 37°16'15"N, 128°46'30"E, col. 6.X.2001, em. 24–26.X.2001, ex Lactuca indica var. laciniata flower, H.-Y. Han et al. (both wings glued on a rectangular card; YSUW08100129; GenBank Acc. Nr. MN445537).
	2. ♀, KOREA: Jeju-do, Jeju-si, Aewol-eup, along rt 1117, col 19.X.2005, em. 23–31.X.2005, ex Lactuca indica var. laciniata flower, H.-Y. Han et al. (both wings glued on a rectangular card; YSUW08100130; GenBank Acc. Nr. MN445538).
*C. difficilis* (Hendel, 1927)	1. ♂, SWITZERLAND: Valais 1689–1950 m, Portes du Soleil, Morgins, 27.VII.2004, H.-Y. Han & K.-E. Ro. (specimen with the abdomen detached; YSUW140201038; GenBank Acc. Nr. MN445539).
*C. guttella* (Rondani, 1870)	1. ♀, SWITZERLAND: Valais 1787–2041 m, Pointe de Bellevue, Morgins, 28.VII.2004, H.-Y. Han & K.-E. Ro (specimen with the abdomen detached; YSUW140201039; GenBank Acc. Nr. MN445540).
*C. hirayamae* (Matsumura, 1916)	1. ♀, KOREA: Gangwon-do, Jeongseon-gun, Nam-myeon, Mt. Mindungsan, from Yupyeong-ri to 1,119 m peak, 37°16'15"N, 128°46'30"E, 24.VI.2005, Han et al. (both wings glued on a rectangular card; YSUW06010914; GenBank Acc. Nr. MN445541).
	2. ♂, KOREA: Gangwon-do, Pyeongchang-gun, Yongpyeon-myeon, S. Valley of Mt. Gyebangsan, 3.X.2003, H.-Y. Han et al. (both wings glued on a rectangular card; YSUW08100131; GenBank Acc. Nr. MN445542).
	3. ♀, KOREA: Gyeongsangbuk-do, Yeongju-si, Sunheung-myeon, Mt. Sobaeksan, Choamsa to Gukmangbong (1421 m), 27.V.2005, H.-W. Byun (both wings glued on a rectangular card; YSUW08100132; GenBank Acc. Nr. MN445543).
*C. loewiana* (Hendel, 1927)	1. ♀, MONGOLIA: Tuv Prov., Tusgalt Valley, Forestry Research-Training Center, Ntn. Univ. of Mongolia, 48°15'37"N 106°51'11"E, 1277 m, 4.VII.2013, H.Y. Han & H.S. Lee (specimen with the abdomen detached; YSUW140201075; GenBank Acc. Nr. MN445544).
	2. ♂, MONGOLIA: Tuv Prov., Tusgalt Valley, Forestry Research-Training Center, Ntn. Univ. of Mongolia, 48°15'37"N 106°51'11"E, 1277 m, 4.VII.2013, H.Y. Han & H.S. Lee (specimen with the abdomen detached; YSUW140201076; GenBank Acc. Nr. MN445545).
	3. ♂, MONGOLIA: Tuv Prov., Tusgalt Valley, Forestry Research-Training Center, Ntn. Univ. of Mongolia, 48°15'23"N, 106°50'23"E, 1522 m, 5.VII.2013, H.Y. Han & H.S. Lee (specimen with the abdomen detached; YSUW140201081; GenBank Acc. Nr. MN445546).
*C. longipennis* Shiraki, 1933, comb. nov. from *Homoeotricha*	1. ♂, RUSSIA: Sakhalin, Yuzhno-Sakhalinsk Vestochka, 46°51'58.3"N, 142°50'54.9"E, 18.VII.2008, H.Y. Han & H.S. Lee (specimen with the abdomen detached; YSUW090915062; GenBank Acc. Nr. MN445547).
*C. luxorientis* (Hering, 1940)	1. ♀, KOREA: Gangwon-do, Jeongseon-gun, Nam-myeon, Mt. Mindungsan, from Yupyeong-ri to 1,119 m peak, 37°16'15"N, 128°46'30"E, 29.VIII.2005, H.-Y. Han et al. (both wings glued on a rectangular card; YSUW090915035; GenBank Acc. Nr. MN445548).
	2. ♂, KOREA: Gangwon-do, Jeongseon-gun, Nam-myeon, Mt. Mindungsan, from Yupyeong-ri to 1,119 m peak, 37°16'15"N, 128°46'30"E, 29.VIII.2005, H.-Y. Han et al. (both wings glued on a rectangular card; YSUW090915036; GenBank Acc. Nr. MN445549).
*C. melaena* (Hering, 1941)	1. ♂, RUSSIA: Primorsky-Krai, Khasansky-District, Barabash, 43°10'46.9"N, 131°28'20.0"E, 22.VI.2008, H.Y. Han & H.S. Lee (specimen with the abdomen detached; YSUW140201105; GenBank Acc. Nr. MN445560).
	2. ♂, RUSSIA: Primorsky-Krai, Nadezhdinsky-District, Vol’no-Nadezhdinskoye, N43°22'31.6”, E132°01'43.1”, 22.VI.2008, H.-Y. Han & H.-S. Lee (specimen with the abdomen detached; YSUW140201106; GenBank Acc. Nr. MN445561).
*C. melanochroa* (Hering, 1941)	1. ♀, KOREA: Gangwon-do, Jeongseon-gun, Nam-myeon, Mt. Mindungsan, from Yupyeong-ri to 1,119 m peak, 37°16'15"N, 128°46'30"E, col. 6.X.2001, em. 26–30.X.2001, ex *Aster ageratoides* Turcz. flower, H.-Y. Han et al. (both wings glued on a rectangular card; YSUW090915039; GenBank Acc. Nr. MN445554).
	2. ♂, KOREA: Gangwon-do, Jeongseon-gun, Nam-myeon, Mt. Mindungsan, from Yupyeong-ri to 1,119m peak, 37°16'15"N, 128°46'30"E, col. 25.IX.2003, em. 13–20.IX.2003, ex *Aster tataricus* L. flower, H.-Y. Han et al. (both wings glued on a rectangular card; YSUW090915040; GenBank Acc. Nr. MN445555).
*C. messalina* (Hering, 1937)	A1. ♂, KOREA: Gangwon-do, Pyeongchang-gun, Yongpyeon-myeon, S. Valley of Mt. Gyebangsan, 5.VIII.2005, H.-Y. Han & H.-S. Lee (both wings glued on a rectangular card; YSUW08100133; GenBank Acc. Nr. MN445550).
	A2. ♀, KOREA: Gangwon-do, Jeongseon-gun, Gohan-eup, Mt.Hambaeksan, Recreation Forest to Manhang-jae, col. 10.X.2003, em 3–6.V.2004 ex *Artemisia* sp. flower, H.-Y. Han & K.-E. Ro (both wings glued on a rectangular card; YSUW08100134; GenBank Acc. Nr. MN445551).
	B1. ♂, KOREA: Gangwon-do, Jeongseon-gun, Gohan-eup, Mt.Hambaeksan, Recreation forest to Manhang-jae, col.10.X.2003, em. 3–6.V.2004, ex *Artemisia* sp. flower, H.-Y. Han & K.-E. Ro (both wings glued on a rectangular card; YSUW090915037; GenBank Acc. Nr. MN445552).
	B2. ♀, KOREA: Gangwondo, Jeongseon, Nammyeon, Mt. Mindungsan, from Yupyeongri to 1,119 m peak, 37°16'15"N, 128°46'30"E, 29.VIII.2005, H.-Y. Han et al. (both wings glued on a rectangular card; YSUW090915038; GenBank Acc. Nr. MN445553).
*C. misella* (Loew, 1869)	1. ♀, HUNGARY: Bdaors, Odvas hg., 18.VI.1991, Merz & Adams (both wings glued on a rectangular card; YSUW94082638; GenBank Acc. Nr. MN445556).
*C. misella* (Loew, 1869)	2. ♀, SWITZERLAND: Valais, Leuk-Rotafen, 46°18'59"N, 7°40'18"E, 640 m, 22.VII.2004, H.-Y. Han & K.-E. Ro (specimen with the abdomen detached; YSUW130901215; GenBank Acc. Nr. MN445557).
	3. ♂, SWITZERLAND: Valais, Leuk-Rotafen, 46°18'59"N, 7°40'18"E, 640 m, 22.VII.2004, H.-Y. Han & K.-E. Ro (specimen with the abdomen detached; YSUW140201041; GenBank Acc. Nr. MN445558
	4. ♀, SWITZERLAND: Valais, Visperterminen-Kreuz, 46°15'17"N, 7°53'52"E, 1500 m, 21.VII.2004, H.-Y. Han & K.-E. Ro (specimen with the abdomen detached; YSUW140201042; GenBank Acc. Nr. MN445559).
*C. paramelaena* sp. nov.	1. Holotype ♂, KOREA: Gyeongsangbuk-do, Bonghwa-gun, Myeongho-myeon, Mt. Cheongnyangsan, 36°46'43.6"N, 128°55”30.8’E, 600 m, 30.VI.2007, H.Y. Han et al. (specimen with the abdomen detached; YSUW090915094; GenBank Acc. Nr. MN445564).
	2. Paratype ♀, RUSSIA: Primorsky-Krai: between Chernyatino and Pokrovk, 43°57'32.7"N, 131°32'24.1"E, 55 m, 26.VI.2008, H.Y. Han & H.S. Lee (both wings glued on a rectangular card; YSUW090915019; GenBank Acc. Nr. MN445565).
	3. Paratype ♂, RUSSIA: Khasansky-District, Kedrovaya Pad, 43°05'09.4"N, 131°35'06.0"E, 22m, 23.VI.2008, H.Y. Han & H.S. Lee (specimen with the abdomen detached; YSUW140201108; GenBank Acc. Nr. MN445566). Paratype.
	4. Paratype ♀, RUSSIA: Khasansky-District, Barabash, 43°10'46.9"N 131°28'20.0"E, 61m, 22.VI.2008, H.Y. Han & H.S. Lee (specimen with the abdomen detached; YSUW090915068; GenBank Acc. Nr. MN445567). Paratype.
*C. producta* (Loew, 1844)	1. ♀, ISRAEL, Golan Heights, Mt. Hermon, 2000 m, 29.V.2000, H.-Y. Han & K.-E. Ro (specimen with the abdomen detached; YSUW130901194; GenBank Acc. Nr. MN445568).
*C. quadriguttata* (Hendel, 1927)	1. ♂, KOREA: Gangwon-do, Jeongseon-gun, Nam-myeon, Mt. Mindungsan, from Yupyeongri to 1,119 m peak, 37°16'15"N, 128°46'30"E, 19.VII.2005, H.-Y. Han et al. (specimen with the abdomen detached; YSUW090915089; GenBank Acc. Nr. MN445569).
	2. ♀, KOREA: Gangwon-do, Pyeongchang-gun, Yongpyeon-myeon, S. Valley of Mt. Gyebangsan, 3.X.2003, H.-Y. Han et al. (specimen with the abdomen detached; YSUW090915090; GenBank Acc. Nr. MN445570).
*C. sabroskyi* (Novak, 1974)	1. ♂, USA: Utah: Grand Co., La Sal Mt. Warner Lake, 7.IX.1992, A.L. Norrbom, ex flower of *Senecio* sp. (1♂, 1♀ from same collecting lot; HAN115; GenBank Acc. Nr. MN445529).
*C. shensiana* (Chen, 1938)	1. ♂, KOREA: Gangwon-do, Wonju-si, Gwirae-myeon, Unnam-ri, col. 13.X.2001, em. 2–16.V.2002, ex *Chrysanthemum boreale*, flower, D.-S. Choi et al. (both wings glued on a rectangular card; YSUW090915041; GenBank Acc. Nr. MN445571).
	2. ♀, KOREA: Gangwondo, Jeongseon, Nammyeon, Mt. Mindungsan, from Yupyeongri to 1,119m peak, 37°16'15"N, 128°46'30"E, col. 6.X.2001, em 9.V.2002, ex *Chrysanthemum makinoi*, flower, H.-Y. Han et al. (both wings glued on a rectangular card; YSUW090915042; GenBank Acc. Nr. MN445572).
	3. ♀, KOREA: Gangwon-do, Samcheok-si, Geunsan-dong, Mt. Geunsan, 37°24'28"N, 129°8'9"E, 4.V.2012, H.-Y. Han et al. (specimen with the abdomen detached; YSUW130901200; GenBank Acc. Nr. MN445573).
*C. sororcula* (Wiedemann, 1830), comb. nov. from *Dioxyna*	4. ♀, JAPAN: Kyushu, Kagoshima-shi, Hirakawa-cho, Goino, 31°27'53"N 130°30'01"E, 66 m, 10.VII.2010 H.-Y. Han & S.-W. Suk (specimen with the abdomen detached; YSUW130901083; GenBank Acc. Nr. MN445574).
	5. ♀, MALAWI: Nyika National Park, Chelinda, 15kmW, 10°35.036’S 33°44.096’E, 2234 m, 31.XII.2009, H.-Y. Han (specimen with the abdomen detached; YSUW130901145; GenBank Acc. Nr. MN445575).
C. sp. near guttella	1. ♀, MONGOLIA: Tuv Prov., Tusgalt Valley, Forestry Research-Training Center, Ntn. Univ. of Mongolia, 48°15'23"N, 106°50'23"E, 1522 m, 5.VII.2013, H.Y. Han & H.S. Lee (specimen with the abdomen detached; YSUW140201077; GenBank Acc. Nr. MN445562).
	2. ♂, MONGOLIA: Tuv Prov., Tusgalt Valley, Forestry Research-Training Center, Ntn. Univ. of Mongolia, 48°15'23"N, 106°50'23"E, 1522 m, 5.VII.2013, H.Y. Han & H.S. Lee (specimen with the abdomen detached; YSUW140201082; GenBank Acc. Nr. MN445563).
*C. spenceri* (Hardy, 1973)	1. ♂, VIETNAM: Lam Dong Prov., Mt. Lang Biang, N of DaLat, 12°02'50.1"N 108°26'26.5"E, 12.XII.2013, H.Y. Han et al. (specimen with the abdomen detached; YSUW140201110; GenBank Acc. Nr. MN445576).

Photographs of pinned specimens were captured with a Panasonic (Osaka, Japan) DMC G5 camera with a Panasonic Lumix 45–175 mm lens and a Raynox (Yoshida Inc., Tokyo, Japan) MSN-202 macro conversion lens. The consecutive digital images in different focal planes (usually 50–100 shots per a single figure) were Z-stacked using Helicon Focus software (Helicon Soft, Ltd., Kharkov, Ukraine). Photographs of live specimens (kept in a glass cage) were taken with a Nikon (Tokyo, Japan) D7000 camera with a macro lens and extension tubes. Photographs of postabdominal structures were taken with a Nikon (Tokyo, Japan) D90 camera mounted on an Olympus (Tokyo, Japan) CX41 compound microscope.

Most of the specimens used in this study are deposited in the Division of Biological Science and Technology, Yonsei University, Wonju, Korea (**YSUW**), and some in the National Institute of Biological Resources, Incheon, Korea (**NIBR**). Abbreviations of the other institutions mentioned in the text are as follows:

**NHMUK**The Natural History Museum, Department of Entomology, London, England, UK;

**IZAS**Institute of Zoology, Academia Sinica, Insect Collection, Beijing, China;

**NIAS**Laboratory of Insect Systematics, National Institute of Agro-Environmental Sciences, Tsukuba, Japan;

**UOPJ**Entomological Laboratory, University of Osaka Prefecture, Osaka, Japan;

**ZMHU**Museum fur Naturkunde der Humboldt Universitat zu Berlin, Bereich Zoologisches Museum, Berlin, Germany.

## Results and discussion

### DNA barcoding and species group recognition

The genus *Campiglossa* is a morphologically homogeneous taxon, and their monophyly has been suggested based on at least two possible synapomorphies: the elongated proboscis and the spinulose phallic preglans area (Korneyev 1999). The published and our present DNA barcoding analyses also indicate that they form a monophyletic group, but together with at least two other genera, *Dioxyna* and *Homoeotricha*.

Smit et al. (2013) performed a DNA barcoding analysis of approximately half of the European tephritids species (42 genera, 135 species, 555 specimens), of which 12 *Campiglossa* and a single *Dioxyna* species were included. In their neighbor-joining tree, five sequences of *Dioxyna bidentis* (Robineau-Desvoidy, 1830) were strongly clustered (90 % bootstrap support) with all other *Campiglossa* sequences, indicating that the genus *Campiglossa* is monophyletic and that *Dioxyna* is merely an aberrant member of *Campiglossa*.

**Figure 1.**
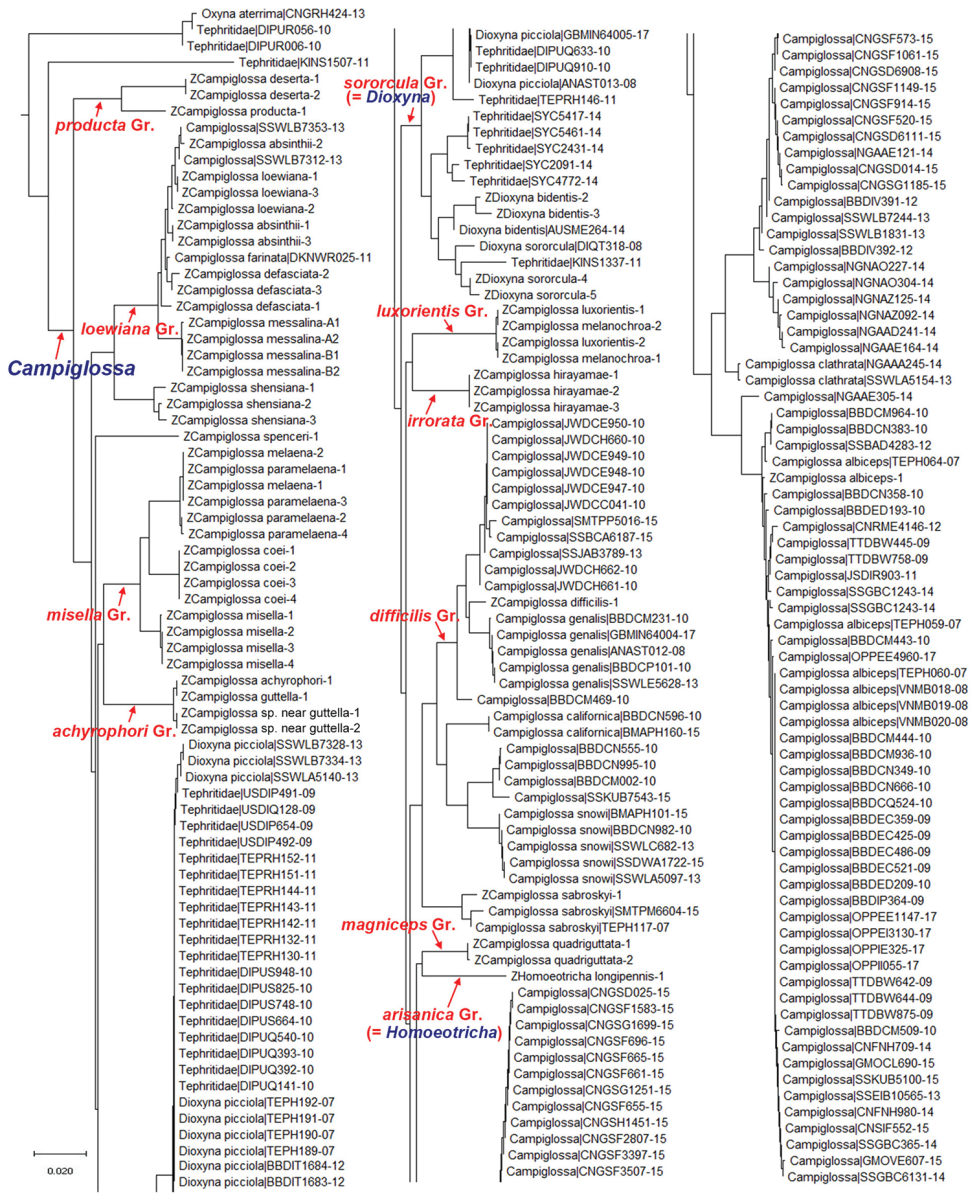
The genus *Campiglossa* portion of the neighbor-joining tree based on the Kimura 2-parameter distances of 7,223 tephritid DNA barcode sequences mostly extracted from BOLD Systems (www.boldsystems.org, as of Jan 2019), including 55 newly obtained *Campiglossa* sequences (names prefixed with Z). All 211 *Campiglossa*, *Homoeotricha*, and *Dioxyna* (regarded to be the genus *Campiglossa*, *sensu lato*, in this study) sequences were recovered as a monophyletic clade in this analysis. Putative species group names (in red) are marked on the respective branches.

As a result of our ongoing DNA barcoding study of the family Tephritidae, we assembled a large dataset of 7,223 individuals, 543 species, and 80 genera publicly available from BOLD systems (www.boldsystems.org), as well as our own dataset of 55 individuals and 26 *Campiglossa* species. The combined dataset contained 7,278 individuals, 543 species and 80 genera. Our simple neighbor-joining analysis recovered a monophyletic cluster of the genera *Campiglossa*, *Dioxyna*, and *Homoeotricha* together (only this portion of the tree is shown in Fig. 1), indicating that the latter two genera should be merged within the genus *Campiglossa*, which has a nomenclatorial seniority. For an updated analysis (Han and Ro, in preparation) of our earlier molecular phylogenetic study of the subfamily Tephritinae (Han et al. 2006), we greatly increased our taxon sampling to include the majority of the *Campiglossa* genus group genera (sensu Norrbom et al. 1999). Our unpublished preliminary molecular analysis grouped the above three genera together, separated from the other closely related genera (i.e., *Desmella* Munro, 1957; *Mesoclanis* Munro, 1938; *Oxyna* Robineau-Desvoidy, 1830; *Scedella* Munro, 1957; and *Tanaica* Munro, 1957), again supporting the expanded concept of the genus *Campiglossa*.

**Figure 2.**
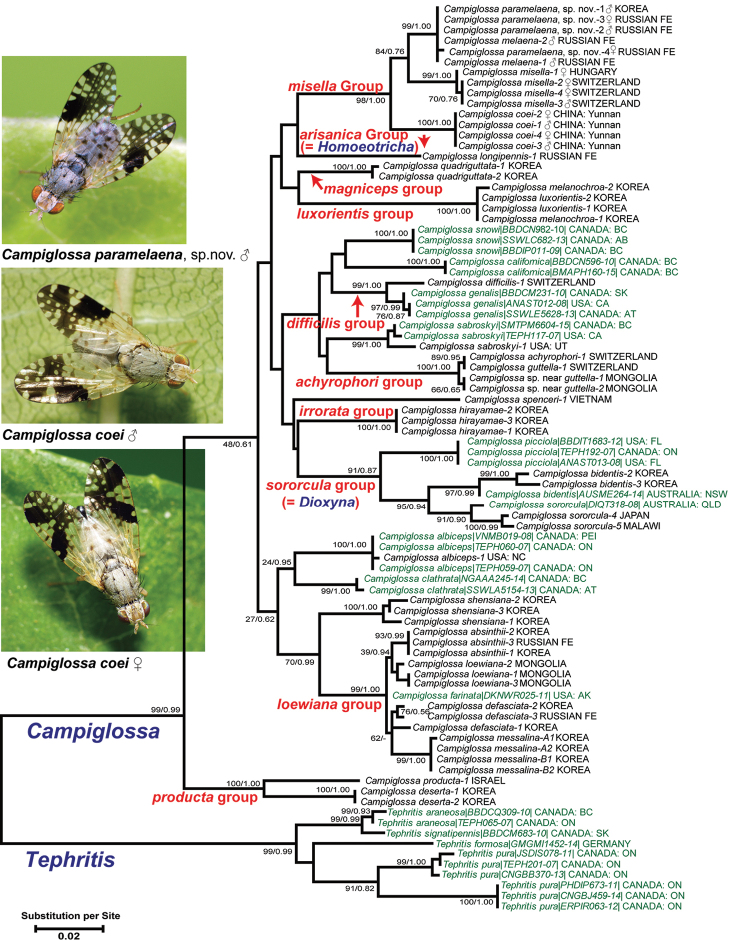
MEGA X analysis produced a maximum-likelihood (ML) phylogram of the 76 selected *Campiglossa* (ingroup) and ten *Tephritis* (outgroup) DNA barcode sequences using the general time reversible model. The first number on each branch is the bootstrap support from ML analysis (pb); the second number represents posterior probability (pp) from Bayesian inference (BI). Samples in green letters were extracted from BOLD systems (www.boldsystems.org).

We also analyzed a scale-down dataset of 32 species and 76 individuals of the genus *Campiglossa* as well as four species and ten individuals of the genus *Tephritis* that is known to be closely related to *Campiglossa* (Norrbom et al. 1999; Korneyev 1999; Merz 1999; Han et al. 2006). These *Tephritis* sequences were used as an outgroup to root the ingroup taxa. Our maximum-likelihood tree (Fig. 2), even though lacking high statistical support on deeper phyletic branches, recognized the following ten major lineages within the *Campiglossa* clade, each of which can be regarded as a monophyletic species group. Recognizing such a group would be an initial step toward establishing a sound classification of this large and confusing genus of Tephritidae.

**The *producta* group** was originally recognized by Merz (1994) including the western and eastern Palaearctic species, *C. producta* (Loew, 1844), and *C. deserta* (Hering, 1939), plus 20 Afrotropical species without listing their specific names. He defined this species group based on the more or less flattened head (approx. as long as wide) and the dark paravertical setae. Our analysis, including the above two species, recovered this group as the basal-most lineage within the *Campiglossa* clade. This result is consistent with Smit et al. (2013) who analyzed DNA barcodes from approximately half of the European tephritid species. *Campiglossa producta* has been reared from the capitula of a wide range of composites, most of which belong to the subfamily Cichorioideae (White 1988). *Campiglossa deserta* was reared by us from the capitula of *Lactuca indica* in Korea (new record).

**The *loewiana* group** includes ca. 30 Holarctic species that have white frontal setulae, and white postocular and posterior notopleural setae (Merz 1994). In our analysis, the five selected species of this group were clearly recovered as a monophyletic group (Fig. 2; pb/pp = 99/1.00).

**The *sororcula* group** was previously known as the genus *Dioxyna*, which is synonymized here with *Campiglossa.* Both our analysis, as well as Smit at al.’s (2013) analysis clearly recovered this group within the *Campiglossa* clade. The only significant morphological differences of *Dioxyna* are their dorsoventrally flattened head as well as rather short apical scutellar setae (not more than 0.25× as long as basal scutellar setae). They are superficially similar to the *producta* group species, especially in having the dorsoventrally flattened head, but the *sororcula* group can be distinguished by their whitish paravertical setae.

**The *irrorata* group**, sensu stricto. In our dataset, this group is only represented by a single species, *C. hirayamae* (Matsumura, 1916), which has a peculiar wing pattern, including the pterostigma with two hyaline spots and the wing margin between apices of veins R_1_ and Cu_1_ with rather regularly arranged nine or ten round hyaline spots. These characteristics seem to be shared by at least the following four species: *C. amurensis* Hendel, 1927; *C. grandinata* (Rondani, 1870); *C. irrorata* (Fallén, 1814); and *C. venusta* Dirlbek & Dirlbeková, 1971. In BOLD Systems (boldsystems.org), our identification attempt using a *C. hirayamae* sequence indeed recovered two closely related species, *C. irrorata* (1.84–2.00 % barcode distance) and *C. grandinata* (1.83–2.15 %). The sequences of these two species were not included in our phylogenetic analyses because they were not open for public download. The name, *irrorata* group, was originally used by Merz (1994), and included a number of distantly related species, but we refined the group more narrowly to include the above species recognized both by DNA barcodes as well as morphology.

**The *achyrophori* group** was originally recognized by Korneyev (1990), listing eight species defined using an identification key. Merz (1994) loosely defined them based on their superficial morphological similarity including the wing with numerous hyaline spots. In our results (Fig. 2), *C. achyrophori* (Loew, 1869), *C. guttella* (Rondani, 1870), and an unidentified species from Mongolia (as C. sp. near guttella) were grouped together as a clear monophyletic clade (Fig. 2; pb/pp = 100/1.00). They are indistinguishable by DNA barcode sequences (0.00–0.26 % barcode distance) but the former two species can only be separated by the relative length of their oviscapes and their host plants (Merz 1994). The unidentified Mongolian species seems to be close to the European *C. guttella* in having a short oviscape, but does have five more distinct longitudinal stripes on the scutum. Since both longer and shorter oviscape individuals exist in the Mongolian specimens roughly sorted as *C. guttella* (Han, personal observation), further study including the female terminalia as well as host relationships is required to clarify their species status.

**The *difficilis* group** was defined by Merz (1992, 1994) based on male genitalic characteristics. He mentioned that there were five species from Palaearctic, Nearctic, and Afrotropical regions without listing their names. Our data at least grouped Palaearctic *C. difficilis* (Hendel, 1927) and Nearctic *C. genalis* (Thomson, 1869) (Fig. 2; pb/pp = 99/1.00). It is interesting to note that *C. difficilis* females are difficult to distinguish from those of *C. misella* (Merz 1994) of the sexually dimorphic *misella* group, which includes six morphologically distinct species (see the next section). Our data indicates that the average barcode distance between the similar looking *C. difficilis* and *C. misella* is 6.27 %, while the average distance among the four morphologically distinct *misella* group species is only 1.64 % (0–2.86 %). Therefore, these observations prove that looks can be deceptive in *Campiglossa*.

**The *luxorientis* group** was originally named by Korneyev (1990) based on *C. luxorientis* (Hering, 1940) and *C. melanochroa* (Hering, 1941) [as *C. dorema* (Hering, 1941)]. Both species show high intraspecific morphological variation as well as remarkable sexual dimorphism in wing patterns (Han 2019). These species could not be separated by DNA barcode sequences, but can easily be distinguished by their morphological characteristics (Fig. 2; Han 2019). They appear to be recently diverged sister species.

**The *magniceps* group** was defined as such by Korneyev (1997) as three species with distinct sexual wing dimorphism [*C. festiva* (Chen, 1938); *C. magniceps* (Hendel, 1927); *C. quadriguttata* (Hendel, 1927)], and were previously transferred by him (Korneyev 1990) to *Campiglossa* from the genus *Gonioxyna* Hendel, 1927. Though only *C. quadriguttata* is included in our analysis, this group appears to be monophyletic based on the long acrophallus of the male glans [illustrated by Korneyev (1990)], which is posited to be a synapomorphy of this group. Their sexually dimorphic male wings appear similar to those of the *arisanica* group (= *Homoeotricha*; see also the next paragraph) especially in having the rounded to angulated anterior wing margin as well as more numerous hyaline spots (Korneyev 1997). The neighbor-joining tree included 7,278 barcode sequences (Fig. 1) clustered these two species groups together, but the maximum likelihood tree, including the selected 76 sequences (Fig. 2) did not group them. Additional genetic markers are needed to test their relationships (Han and Ro, in preparation).

**The *arisanica* group** was previously known as the genus *Homoeotricha*, which is here synonymized with *Campiglossa.* Our DNA barcoding analyses recovered *C. longipennis* Shiraki, 1933, within the *Campiglossa* clade. This species closely resembles *C. arisanica* (Shiraki, 1933), which is the type species of the East Asian genus *Homoeotricha* (the senior author examined the holotype ♀ of *C. longipennis* and the syntype ♂♀ of *C. arisanica* in NIAS). In addition to these two species, four other species are currently listed under this genus (Norrbom et al. 1999). They appear similar to the *magniceps* group species in having sexually dimorphic male wings (see the above paragraph), but the following possible synapomorphies (extracted from Korneyev 1993) differentiate the *arisanica* group: 1) vein R_2+3_ undulate; 2) labella longer than peristomal cavity, expanded in a leaf-like fashion in males; and 3) male genitalia with short and flattened sclerite around opening of acrophallus.

**The *misella* group** is named and reviewed in detail below.

### The *misella* group of the genus *Campiglossa*

Our DNA barcoding analyses (Figs 1, 2) recovered a closely related group of four species (*C. coei*, *C. misella*, *C. paramelaena* sp. nov., and *C. melaena*; average DNA barcode distance 1.64 %, range 0.00–2.86 %), all of which show close morphological resemblance each other. Based on their morphological characteristics, especially the large dark mid-anterior wing marking in males, we recognized two further members, *C. pishanica* and *C. propria* from China, both of which are only known from male specimens.

Merz (1994) previously placed *C. misella* in the *irrorata* group, sensu lato, based on a few male genitalic characteristics, but this species group was not supported in Smit et al.’s (2013) barcoding analysis, which included both *C. misella* and *C. irrorata* (barcode distance approximately 5 %). Our analysis, including the *misella* group and the *irrorata* group, sensu stricto (represented by *C. hirayamae*) did not support their close relationship either (Figs 1, 2; barcode distance of 4.74 %).

**Diagnosis.** Members of the *misella* group can be diagnosed as follows, including the remarkable sexually dimorphic wing pattern: ***Head*** with paravertical and genal setae whitish. ***Thorax*** with both notopleural setae dark; apical scutellar setae at most half as long as basal setae; anepisternum with upper seta strong, dark, but lower seta approx. half as long, whitish; katepisternal seta strong, dark; anepimeral seta strong, whitish. ***Legs*** with both mid and hind coxal setae whitish. ***Male wing*** (except for some European populations of *C. misella* that show small sexual wing dimorphism) with large dark mid-anterior marking (roughly elliptic to inverted triangular shape; e.g., Fig. 4A, C, G) usually covering mid-anterior 1/3 to center of wing. ***Abdominal tergites*** 3–5 in male and 3–6 in female each with pair of brown to dark brown submedian spots (e.g., Fig. 4I, K). ***Male genitalia*** with short proctiger; epandrium plus surstyli oval in caudal view, with posteriorly serrate lateral surstylar flange; preglans area of phallus strongly spinulose; glans without subapical lobe; tube-like acrophallus highly pronounced with apicodorsal opening, approx. half as long as glans; ejaculatory apodeme large, fan-shaped. ***Female postabdomen*** with oviscape cone shaped, dorsoventrally flattened; posterior 3/4 area of eversible membrane densely covered with anteriorly directed triangular spinules; aculeus elongated, dorsoventrally flattened, apically gradually pointed, apex with pair of tiny subapical teeth; two similar sized dark brown spermathecae, each with elliptical apical receptacle with transverse papillae and narrow basal neck; spermathecal duct transparent.

**Distribution.** All the recognized species of the *misella* group are distributed in East Asia including Nepal, China, the Russian Far East, and Korea, but the widespread *C. misella* extends its distribution to Central Asia and to Europe.

**Biology.***Campiglossa misella* is the only species with known biology. White (1988) reported that they usually attack the flowering spikes of *Artemisia vulgaris*, inducing a stem gall in the first generation and developing in the capitula in the second generation in the U.K. (see the Biology section of *C. misella*).

**Remarks.** In addition to the large mid-anterior wing marking, the position of the crossvein R-M is more apically placed in males of all three species measured both sexes (male vs. female vein M ratios of *C. coei* 0.4–0.45 vs. 0.62–0.76; *C. misella*, 0.26–0.28 vs. 0.4–0.49; *C. paramelaena* sp. nov., 0.29–0.43 vs. 0.41–0.53). Such a structural modification seems to be associated with the male wing pattern modification of the *misella* group. We posit that the large mid-anterior dark marking with associated structural modification present only in males is a good candidate for a morphological synapomorphy of this species group. Interestingly, the wing cell r_1_ of *C. propria* (Chen, 1938) male is further modified (see the Diagnosis of *C. propria* and Fig. 10F).

Since the females of the *misella* group do have more typical *Campiglossa* wing patterns and there are a good number of *Campiglossa* species currently known only by females, there might be some more species of the *misella* group not recognized in this study. A further survey of East Asian *Campiglossa* species in conjunction with DNA barcoding analyses is required.

### Key to the species of the *misella* group of the genus *Campiglossa* (an asterisk (*) denotes likely members)

**Table:** 

1	Legs entirely yellow-brown (Fig. 4A)	**2**
–	Legs dark (Fig. 7G) or at least with dark femora (Fig. 4H)	**3**
2	Width of cell r_1_ measured on axis of crossvein R-M as wide as or slightly wider than cell r_2+3_ (as in Fig. 10E-a)	***C. coei***
–	Width of cell r_1_ measured on axis of crossvein R-M approx. twice as wide as cell r_2+3_ (Fig. 10F-a)	***C. propria*** * ♂
3	Scutum dark brown (Fig. 7H); wing cell r_2+3_ with posteroapical hyaline spot (Fig. 7I-a)	***C. melaena***
–	Scutum ash-grey (Fig. 7B); wing cell r_2+3_ without posteroapical hyaline spot (Fig. 7D-a)	**4**
4	Cell br posterior to fork of vein Rs hyaline (Fig. 7C-a)	***C. paramelaena* sp. nov.**
–	Cell br posterior to fork of vein Rs with dark area (Fig. 4G-a, J-a)	**5**
5	Cell r_1_ posterior to pterostigma with two hyaline spots (Fig. 10E-b); apical 1/4 of cell dm with only posterior hyaline spot (Fig. 10E-c) basal 3/4 of cell dm almost entirely hyaline	C. pishanica* ♂
–	Cell r_1_ posterior to pterostigma with three hyaline spots (Fig. 4G-b); apical 1/4 of cell dm with anterior and posterior hyaline spots (Fig. 4J-b); basal 3/4 of cell dm with dark background and 2–3 large hyaline spots	***C. misella***

#### Campiglossa coei

Taxon classificationAnimaliaDipteraTephritidae

(Hardy)

FBEF9957-A3F6-5D22-B6F4-6AC8C9BC681F

Figs 2 , 4A–E , 5A–G , 10A, B

Tephritis coei Hardy, 1964: 164 (Type-locality: NEPAL, Taplejung Dist., N of Sangu, above river bank, ca. 5000 ft, holotype ♂, NHMUK); Wang 1998: 291, 294 (in the East Asian Tephritis key; diagnosis, new Chinese record – 2♂ from Yunnan Province).
Campiglossa coei : Korneyev 1990: 444 (new combination), 2004: 8 (erroneous synonymy with C. misella); Norrbom et al. 1999: 109 (in the world tephritid catalog); Korneyev and Ovchinnikova 2004: 546 (erroneous synonymy with C. misella).
Campiglossa favillacea Ito, 2011: 29 (Type-locality: NEPAL, Taplejung Dist., Kharu Pokhar, 3,000 m, holotype ♂, UOPJ – examined, Fig. 10A, B), syn. nov.

##### Material examined.

Type series of *C. favillacea* Ito, 2011 (UOPJ; Fig. 10A, B): NEPAL: Taplejung: Kharu Pokhar, 3,000 m, 17.VII.1962, T. Yasuda, holotype ♂ of *C. favillacea*; Ilam: Phikol, 1,460 m, 19.IV.1962, T. Yasuda paratype 2♀, of *C. favillacea*. CHINA: Yunnan, Mengsong, Manlvcunhanzudazhai, small hilltop, 22°07'44.0"N, 100°28'51.7"E, 1690 m, 12.VII.2011, H.Y. Han and S.W. Suk, 72♂, 42♀ (YSUW); Yunnan, Mengsong, Bengangxizhai, in forest, 22°10'34.5"N, 100°35'06.8"E, 1725 m, 11.VII.2011, H.Y. Han and S.W. Suk, 1♀ (YSUW).

**Figure 3.**
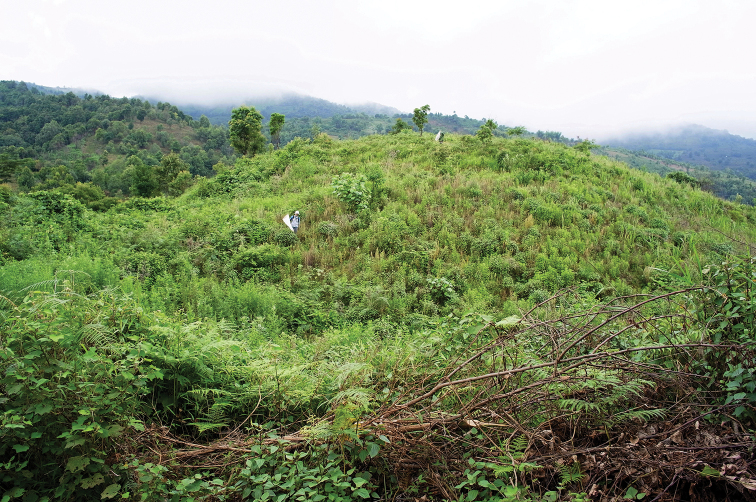
The habitat of *Campiglossa coei*. CHINA: Yunnan, Mengsong, Manlvcunhanzudazhai, small hilltop, 22°07'44.0"N, 100°28'51.7"E, 1,690 m, 12 July 2011. Many more than 100 individuals of *C. coei* were collected along with at least ten other species of the subfamily Tephritinae.

##### Diagnosis.

**Figure 4.**
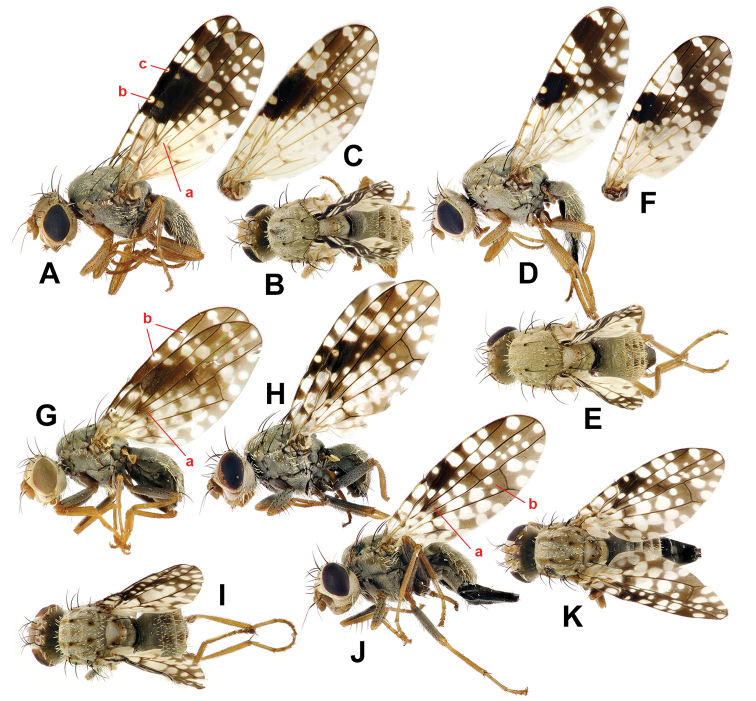
**A–F***Campiglossa coei***A** male, lateral view **B** male, dorsal view **C** male wing **D** female, lateral view **E** female, dorsal view **F** female wing **G–K***C. misella***G** male, lateral view **H** male, lateral view **I** male, dorsal view **J** female, lateral view **K** female, dorsal view.

This light-colored species can be diagnosed by the following characteristics. ***Head*** largely yellow-brown with grey upper occiput. ***Thorax*** with scutum entirely matte whitish grey without any outstanding dark spots or stripes; scutellum mostly matte whitish grey but ca. apical 1/3 yellow-brown. ***Legs*** entirely yellowish brown without any dark marking; fore femur with six or seven strong, brown postero-ventral setae. ***Wing*** with basal area (basal 1/3 anteriorly and basal 1/2 posteriorly) largely hyaline with only few small dark spots, especially cell br with area posterior to fork of vein Rs completely hyaline (Fig. 4A-a); male with large dark mid-anterior marking covering from mid-anterior 1/3 to posterior end of crossvein R-M; pterostigma dark brown with large round hyaline spot in both sexes (Fig. 4A-b; in the other *misella* group species this spot tends to be smaller or missing in male); cell r_1_ posterior to pterostigma with two large hyaline spots (sometimes with tiny additional basal spot, Fig. 4A-c) in male and three large hyaline spots in female; cell r_2+3_ without posteroapical hyaline spot. ***Abdomen*** matte whitish grey with tergites 3–5 in male and 3–6 in female each with pair of pale brown submedian spots; oviscape shiny dark brown, as long as three preceding segments.

This species appears similar to *C. pishanica* (with only males known) but the latter species can be readily separated by the dark femora and more extensive mid-anterior wing marking with pterostigma completely dark (Figs 4A, C vs. 10E).

**Figure 5.**
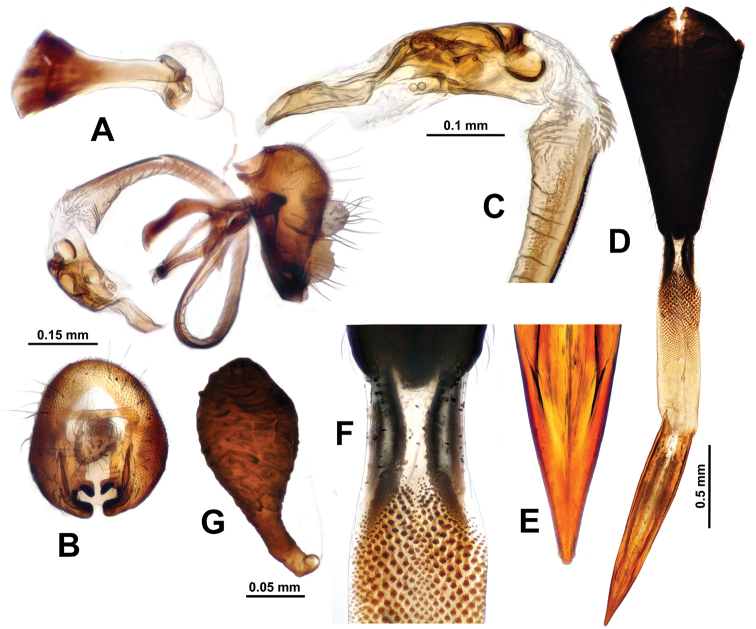
Genitalia of *Campiglossa coei***A** epandrial complex, lateral view **B** epandrial complex, caudal view **C** glans and preglans of distiphallus **D** female postabdomen with aculeus and eversible membrane pulled out, ventral view **E** magnified view of aculeus tip **F** magnified view of oviscape and eversible membrane **G** spermatheca.

##### Description.

***Body*** (Fig. 4A–E) predominantly matte whitish grey; setae mostly brown to dark brown but some white; setulae mostly white but some brown to dark brown; wing length 4.0–4.3 mm; thorax length 1.5–1.8 mm.

***Head*** yellow-brown with whitish pruinosity except for dark brown ocellar triangle and grey upper occiput; head ratio 0.78–0.90, frons-head ratio 0.46–0.50, eye ratio 0.71–0.77, gena-eye ratio 0.17–0.23, antenna-head ratio 0.40–0.44, arista-antenna ratio 1.3–1.6; vertex yellow-brown; dark brown inner vertical seta approx. as long as longest diameter of eye; outer vertical seta white, 0.4× inner vertical seta; post ocellar seta white, 0.4× post ocellar seta; paravertical seta white, 0.7–0.8× post ocellar seta; ocellar seta dark brown, 3.3–4.0× ocellar triangle length; frons almost bare with frontal angle ca. 115 degree; with two dark brown frontal setae; white posterior orbital seta 0.6× dark brown anterior orbital seta; scape and pedicel yellow-brown with short brown setulae; first flagellomere 1.4–1.8× pedicel length, apically rounded, yellow-brown; arista entirely short pubescent, brown except yellow-brown basal area; face yellow-brown without distinct antennal groove; parafacial 0.4× as wide as first flagellomere; facial ridge with fine pale yellow setulae; gena with strong white genal seta and relatively long white setulae; postgena swollen with strong white postgenal seta and relatively long white setulae; postocular setae with two thick white setulae plus over ten shorter brown setulae, extended 0.5× distance from upper eye margin to lower eye margin; supracervical setae white; mouthparts geniculated with yellow-brown setulose labella; palpus with brown setulae apically and white setulae on remaining area.

***Thorax*** largely dark brown ground color with very heavy whitish pruinosity, generally appearing matte whitish grey; postpronotal lobe with single dark brown seta, yellow-brown in ground color, but appearing similar color as nearby areas due to heavy whitish pruinosity; scutum matte whitish grey with five faint brownish longitudinal bands traceable in clean specimens; two pairs of white scapular setae; acrostical setae widely separated each other, situated midway between levels of intra-alar setae and postsutural supra-alar setae; post-alar setae same level as intra-alar setae; dorsocentral setae same level as or slightly lower than transverse suture; presutural supra-alar setae approximately the same level as anterior notopleural setae; two notopleural setae dark brown with posterior seta0.5× anterior seta; scutellum mostly matte whitish grey but ca. apical 1/3 yellow-brown, slightly convex, almost bare except marginal tiny white setulae; basal scutellar setae more or less parallel, 2.3–3.5× as long as scutellum; apical scutellar setae crossed near apex, 0.9–1.3 as long as scutellum; pleura largely matte whitish grey; proepisternum with 3–5 white setulae; anepisternum matte grey with posterior 2/3 white setulose, with one strong dark brown seta and one half as long white seta ventral to it; katepisternum matte grey with a strong dark brown seta, upper area sparsely with short white setulae and lower area with long white setulae; mediotergite matte grey.

***Legs*** entirely yellow-brown with slight grey pruinosity and brown to dark brown setae and setulae; fore coxa anteriorly with white setulae, posteriorly bare; mid coxa anteriorly with few long white setulae, posteriorly bare; hind coxa with strong white lateral seta, posteriorly largely membranous; front femur with six or seven strong brown posteroventral setae; tibiae and tarsi entirely yellow-brown; midtibial spur dark brown, 1.2–1.4 as long as tibial width.

***Wing*** (Fig. 4A, C, D, F) hyaline with brown to dark brown pattern; area around pterostigma with sexual dimorphism (see next paragraph); cells bc, bm, bcu, alula, anal lobe almost entirely hyaline; cell c mostly hyaline with narrow brown to faint brown medial longitudinal band; pterostigma with distinct hyaline spot in both sexes (Fig. 4A-b); cell r_2+3_ mostly without apical hyaline spot but with two large subapical spots often coalesced, one or two large hyaline spots posterior to two large r_1_ spots; cell br with basal 3/5 area almost hyaline, apically dark brown with two or three hyaline spots posteriorly coalesced; cell r_4+5_ with single large apical spot and 8–12 variably sized hyaline spots; cell dm with basal 2/3 almost hyaline, apically dark brown with 4–7 variably shaped hyaline spots; cell m with basal 3/4 almost hyaline, apically dark brown with 1–3 variably shaped hyaline spots. Wing-thorax ratio 2.4–2.5; subcostal to costa ratio 0.43–0.53; cell r_1_-r_2+3_ ratio 2.7–3.3; cell r_4+5_-r_2+3_ ratio 0.58–0.73. R_4+5_ bare.

***Wing dimorphism.*** Male (Fig. 4A, C) with cell r_1_ with two large hyaline spots apical to pterostigma (rarely tiny additional spot anteriorly; Fig. 4A-c); large, more or less elliptic dark brown mid-anterior marking traceable covering pterostigma, cell r_1_ well beyond pterostigma, approx. basal 1/3 to 2/3 of cell r_2+3_, and anterior areas of cells br and r_4+5_ near crossvein R-M; vein M ratio 0.40–0.45. Female (Fig. 4D, F) – cell r_1_ with three large hyaline spots apical to pterostigma; dark brown mid-anterior marking, if traceable, much smaller, or not wider than pterostigma; vein M ratio 0.62–0.76.

**Figure 6.**
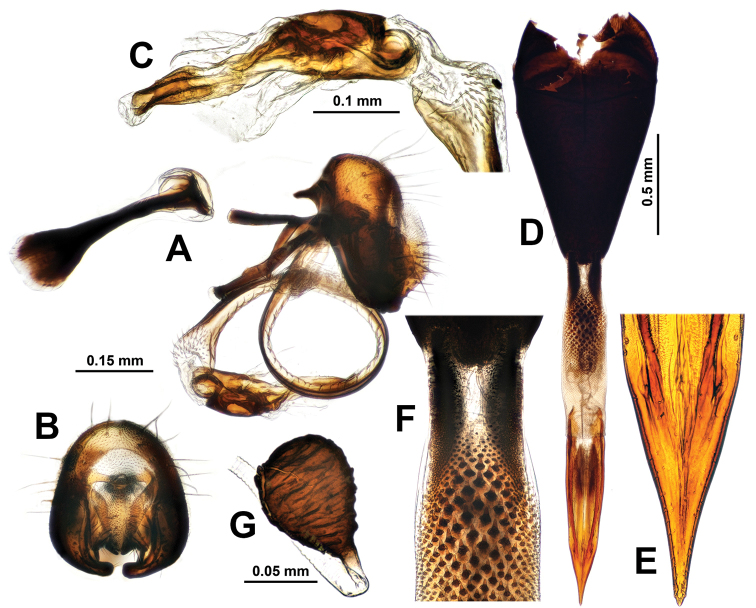
Genitalia of *Campiglossa misella***A** epandrial complex, lateral view **B** epandrial complex, caudal view **C** glans and preglans of distiphallus **D** female postabdomen with aculeus and eversible membrane pulled out, ventral view **E** magnified view of aculeus tip **F** magnified view of oviscape and eversible membrane **G** Spermatheca.

***Male abdomen.*** Preabdomen slightly longer than wide, almost entirely matte pale grey; tergites 2–5 with white setulae, but tergite 5 also with 4–7 dark brown marginal setae; tergites 3–5 each with pair of pale brown submedian spots. Postabdomen (Fig. 6A–C) with proctiger short, 0.4× as long as epandrium in lateral view, microtrichosae, lower half with numerous yellow-brown setae; epandrium plus surstyli oval in caudal view; epandrium dark brown with long yellow-brown to brown setae, microtrichosae; lateral surstylar flange posteriorly serrate, with its basal width approx.1/3 as long as epandrial complex height; medial surstylus with lateral prensiseta approx.2/3 as long as medial prensiseta; preglans area of phallus strongly spinulose; glans without subapical lobe; tube-like acrophallus highly pronounced with apicodorsal opening, approx. half as long as glans; ejaculatory apodeme large, fan-shaped.

***Female abdomen*.** Preabdomen slightly longer than wide, almost entirely matte grey; tergites 2–6 with white setulae, and tergite 6 especially with 4–7 dark brown marginal setae; tergites 3–6 each with pair of pale brown submedial spots. Postabdomen (Fig. 6D–G) with shiny dark brown oviscape approx. as long as three preceding segments; oviscape densely with dark brown setulae but without any macrosetae, 1.8× longer than wide, cone shaped, dorsoventrally flattened; eversible membrane with taeniae approx.1/4 as long as total length of membrane; posterior 3/4 area of eversible membrane densely covered with anteriorly directed triangular spinules; spinules largest in area behind taeniae; aculeus elongated, dorsoventrally flattened, 5.5× longer than wide with apical 1/3 gradually pointed, apex with pair of tiny subapical teeth; two similar sized dark brown spermathecae, each with elliptical apical receptacle with transverse papillae and 3/5 as long narrow basal neck; spermathecal duct transparent.

##### Distribution.

Nepal, China (Yunnan).

##### Remarks.

The male wing pattern of *C. coei* is atypical for the genus *Campiglossa* (Fig. 4A, C), and that is probably why this species, based on a single male specimen, was originally classified as *Tephritis* by Hardy (1964). Since then, Wang (1998), under this name, recorded two males from Yunnan, China. More recently, Ito (2011) described a new species (*C. favillacea* syn. nov.) based on the male holotype (from the type locality of *C. coei*) and two female paratypes (Fig. 10A, B), but he did not mention their wing dimorphism in the description. We, fortunately, were able to collect over a hundred male and female specimens from China, showing a remarkable sexual wing dimorphism (Fig. 4A, C vs. Fig. 4D, F). Most of the specimens were collected along with at least ten other species of the subfamily Tephritinae from a small hilltop in Yunnan, China (Fig. 3; Mengsong, Manlvcunhanzudazhai, 22°07'44.0"N, 100°28'51.7"E, 1690 m, 12 July 2011). This hilltop appears to be a temporary Tephritinae hot spot due to the clearing of a small forest patch.

#### Campiglossa misella

Taxon classificationAnimaliaDipteraTephritidae

(Loew)

5B77CD35-419B-514E-AD70-19DF951ED515

Figs 4G–K , 6A–G

Oxyna misella Loew, 1869: 19 (Type-locality: RUSSIA, Sarepta [Volgograd Region]. Syntype ♂♀, ZMHU. Inference of holotype by White 1986: 152, invalid; l.c. Norrbom et al. 1999: 112).
Tephritis lusoria Nowicky, 1869: 145 (Type-locality: UKRAINE, “Podolu, Sinkowie”; and Skale [Skala Podilska]. Syntype ♂, ZMHU, inference of holotype by White 1986: 152 (invalid; depository of other syntypes unknown); l.c. Norrbom et al. 1999: 112).
Paroxyna kunlunica Wang, 1996: 185 (Type-locality: CHINA, Yecheng, Xinjiang. Holotype ♂, IZAS); Wang 1998: 267 (new synonym of C. misella).
Campiglossa roscida Ito, 2011: 28 (Type-locality: NEPAL, Taplejung Dist., Walungchung Gola, 3,350 m. Holotype ♀, UOPJ – examined, Fig. 10C, D), syn. nov.
Campiglossa misella : Korneyev 1990: 443 (new combination, redescription); Norrbom et al. 1999: 112 (in the world Tephritidae catalog); Korneyev and Kameneva 1993: 44 (host plants); Wang 1998: 255, 267 (in the East Asian Campiglossa key, diagnosis); Korneyev 2004: 8 (taxonomic notes and erroneous synonymy of Tephritis coei and T. pishanica – see Remarks); Korneyev and Ovchinnikova 2004 (in the Russian Far East Tephritidae key); Smit et al. 2013: 297 (DNA barcoding analysis).
Paroxyna misella : Hendel 1927: 155, X-2 (description, wing photograph of a syntype male); White, 1988: 5, 50 (biology, diagnosis, in the British Paroxyna key).

##### Material examined.

HUNGARY: Bdaors, Odvas hg., 18.VI.1991, B. Merz and Adams, 1♀ (YSUW). ITALY: Aosta, St. Pierre, M. Torrette, 800–850 m, 22.IV.2003, B. Merz and F. Amiet, 1♂ (YSUW). NEPAL: Taplejung: Walungchung Gola, 3,350 m, 14.VI.1962, T. Yasuda, holotype ♂ of *C. roscida* (UOPJ; Fig. 10C, D). SWITZERLAND: Valais 642 m, St. German/Brüke, 3.VIII.1998, B. Merz and G. Bächli, 1♀; Valais, Leuk-Rotafen, 46°18'59"N, 7°40'18"E, 640 m, 22.VII.2004, H.Y. Han and K.E. Ro, 1♂ 1♀ (YSUW); Valais, Visperterminen-Kreuz, 46°15'17"N, 7°53'52"E, 1500 m, 21.VII.2004, H.Y. Han and K.E. Ro, 2♀ (YSUW). KYRGYZSTAN: S-Issik-Kul nr. Barskaun vill., 31.VII.1995, S.V. Ovchinnikov, 1♀ (YSUW); Telash Mt. r./ N slope, Ara-Bijik rav, 2300 m, 4.VII.1998, D. Milko, 1♂ (YSUW).

##### Diagnosis.

Males of *C. misella* usually have distinct sexually dimorphic wing patterns [e.g., Fig. 4G from Kyrgyzstan is almost identical to the male syntype photograph by Hendel (1927)] but some European populations seem to show slight sexual dimorphism (e.g., Fig. 4H from Switzerland). More extensive survey is required to understand their variation, but they could still be readily diagnosed even based on our limited samples. ***Head*** largely yellowish brown with grey upper occiput. ***Thorax*** with scutum entirely ash-grey with five brownish longitudinal stripes (Fig. 4I, K); bases of acrostichal, dorsocentral, intra-alar, basal scutellar setae dark brown; scutellum ash-grey with lateral margins brown, apex yellowish brown; ***Legs*** with femora largely dark grey except for yellowish brown apices (Fig. 4G, H, J), but tibiae and tarsi yellowish brown; fore femur with six or seven dark brown posteroventral setae. ***Wing*** with basal half largely with dark spots, especially cell br posteroapical to fork of vein Rs with dark brown rectangular area (approx. twice as wide as long; Fig. 4G-a, J-a); male often with large dark mid-anterior marking covering from mid-anterior 1/3 to posterior end of crossvein R-M (Fig. 4G); pterostigma almost completely dark brown in such sexually dimorphic male (Fig. G), but with large hyaline spot in minimally dimorphic male (Fig. 4H), and female (Fig. 4J); cell r_1_ apical to pterostigma with three large hyaline spots with 1^st^ and 3^rd^ spots much smaller than middle one in dimorphic male (Fig. 4G-b), but with three large similarly sized hyaline spots in female (Fig. 4J) or minimally dimorphic male (Fig. 4H); cell r_2+3_ without posteroapical hyaline spot. ***Abdomen*** ash-grey with tergites 3–5 in male and 3–6 in female each with pair of brown submedian spots; oviscape shiny dark brown, as long as four preceding segments.

##### Distribution.

Europe, Central Asia, China (Xinjian, Shanxi, Sichuan, Xizang, Yunnan), Nepal.

##### Biology.

This is the only species of the *misella* group with host feeding biology known. Interestingly, White (1988) reported that this species usually attacks the flowering spike of *Artemisia vulgaris*, inducing a stem gall in the first generation and developing in the capitula in the second generation in the UK. In addition to *Ar. vulgaris*, Korneyev and Kameneva (1993) listed *Ar. santolinifoliae and Ar. dracunculus* as their host plants in Central Asia (Kazakhstan).

##### Remarks.

We resurrected *C. coei* and *C. pishanica* from the synonymy of *C. misella* by Korneyev (2014). Our study indicates that *C. coei* is a valid species (Figs 1, 2). *Campiglossa pishanica* is somewhat similar to *C. misella* in having the dark femora and the large mid-anterior wing marking, but *C. pishanica* has the following characteristics that, we posit, are beyond the variation range of the *C. misella* wing pattern (Figs 4G vs. 10E): cell r_1_ apical to pterostigma with two hyaline spots instead of 3, basal 3/4 of cell dm almost hyaline, and anal lobe hyaline. See also the Remarks of *C. pishanica* for further discussion.

#### Campiglossa paramelaena sp. nov.

Taxon classificationAnimaliaDipteraTephritidae

FE17C433-902A-54FB-AB67-1BABB1966A88

http://zoobank.org/0B2A4DE8-E854-4722-8AB8-374B36D68E12

Figs 5A–F , 8A–G

##### Type material.

***Holotype*** ♂: KOREA: Gyeongsangbuk-do, Bonghwa-gun, Myeongho-myeon, Mt. Cheongnyangsan, 36°46'43.6"N, 128°55'0.8"E, 600 m, 30.VI.2007, H.Y. Han et al. (NIBR). ***Paratypes***: RUSSIA: Primorsky-Krai: between Chernyatino and Pokrovk, 43°57'32.7"N, 131°32'24.1"E, 55 m, 26.VI.2008, H.Y. Han and H.S. Lee, 3♂ 3♀; Khasansky-District, Kedrovaya Pad, 43°05'09.4"N, 131°35'06.0"E, 22 m, 23.VI.2008, H.Y. Han and H.S. Lee, 1♂; Khasansky-District, Barabash, 43°10'46.9"N, 131°28'20.0"E, 61 m, 22.VI.2008, H.Y. Han and H.S. Lee, 1♀; Ussuriysk, 43°47'05.4"N, 132°01'37.8"E, 19 m, 26.VI.2008, H.Y. Han and H.S. Lee, 1♀. All paratypes in YSUW.

##### Etymology.

The specific epithet is derived from the closely related species *melaena* prefixed with *para*.

##### Diagnosis.

**Figure 7.**
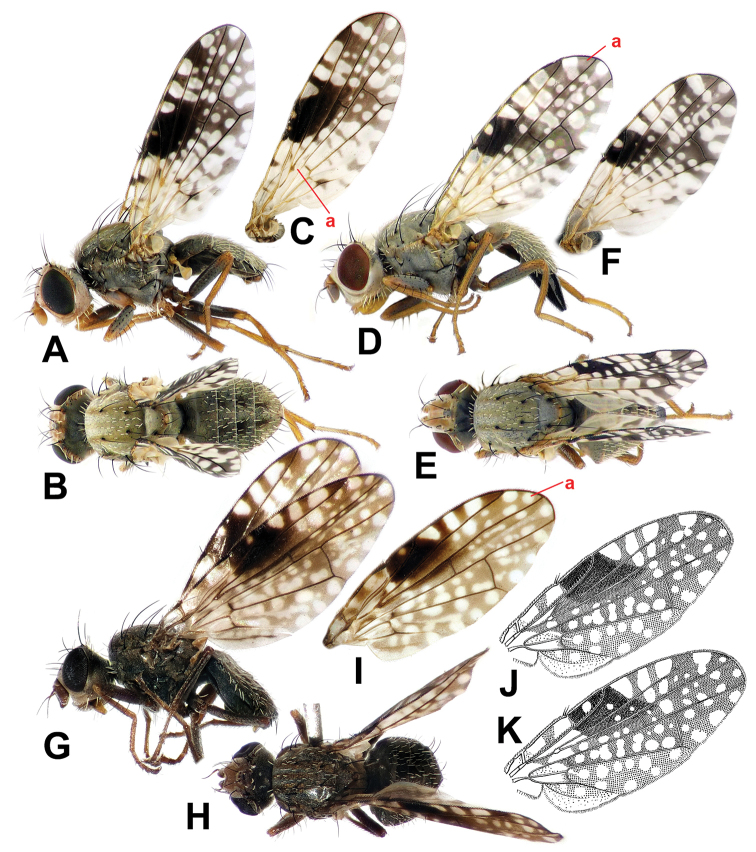
**A–E***Campiglossa paramelaena* sp. nov. **A** male, lateral view **B** male, dorsal view **C** male wing **D** female, lateral view **E** female, dorsal view **F** female, wing **G–K***C. melaena***G** male, lateral view **H** male, dorsal view **I** male, wing **J** holotype male, wing **K** female, wing **J, K** Reproduced from Korneyev and Ovchinnikova (2004) with permission from Valery Korneyev.

This new species can be diagnosed by the following characteristics. ***Head*** largely yellowish brown with grey upper occiput. ***Thorax*** with scutum entirely ash-grey with five faint brownish longitudinal stripes (Fig. 7B, E); bases of acrostichal, dorsocentral, intra-alar, basal scutellar setae dark brown; scutellum ash-grey with apex yellowish brown; ***Legs*** with femora largely dark grey except for yellowish brown apices (Fig. 4G, H, J), but tibiae and tarsi yellowish brown; fore femur with six or seven dark brown posteroventral setae. ***Wing*** with basal area (basal 1/3 anteriorly and basal 1/2 posteriorly) largely hyaline with only few small dark spots, especially cell br with area posterior to fork of vein Rs completely hyaline (Fig. 7C-a); male with large dark mid-anterior marking covering from pterostigma to posterior end of crossvein R-M; male pterostigma almost completely dark brown, at most with tiny hyaline spot (Fig. 7C-b); female pterostigma with large round hyaline spot (Fig. 7D-a); cell r_1_ posterior to pterostigma with three large hyaline spots in both sexes; cell r_2+3_ without posteroapical hyaline spot (Fig. 7C-c, D-a). ***Abdomen*** ash-grey with tergites 3–5 in male and 3–6 in female each with pair of brown submedian spots; oviscape shiny dark brown, as long as three preceding segments.

*Campiglossa paramelaena* sp. nov., appears similar to *C. misella* but the former species can be readily separated by the almost hyaline basal area of the wing, and the area posterior to the fork of vein Rs in particular is completely hyaline while the latter species has a distinctly dark spot on that area (Fig. 7C-a vs. Fig. 4G-a, J-a).

##### Description.

***Body*** (Fig. 7A–F) predominantly ash-grey; setae mostly dark brown but some white; setulae mostly white but some dark brown; wing length 3.0–3.8 mm; thorax length 1.2–1.5 mm.

***Head*** yellow-brown with whitish pruinosity except for dark grey ocellar triangle and upper occiput; head ratio 0.85–0.92, frons-head ratio 0.47–0.53, eye ratio 0.75–0.83, gena to eye ratio 0.17–0.22, antenna-head ratio 0.41–0.46, arista-antenna ratio 1.3–1.7; vertex yellow-brown; dark brown inner vertical seta approximately as long as longest diameter of eye; outer vertical seta white, 0.4× inner vertical seta; post ocellar seta white, 0.3–0.4× post ocellar seta; paravertical seta white, 0.7–0.9× post ocellar seta; ocellar seta dark brown, 3.0–3.5× ocellar triangle length; frons almost bare with frontal angle 110–115 degree; with two dark brown frontal setae; white posterior orbital seta 0.6–0.8× dark brown anterior orbital seta; scape and pedicel yellow-brown with short dark brown setulae; first flagellomere 1.5–2.1× pedicel length, apically rounded, yellow-brown but with greyish tinge in some individuals; arista entirely short pubescent, dark brown except yellow-brown basal area; face yellow-brown without distinct antennal groove; parafacial 0.4–0.5× as wide as first flagellomere; facial ridge with fine pale yellow setulae; gena with strong white genal seta and relatively long white setulae; postgena swollen with strong white postgenal seta and relatively long white setulae; postocular setae with two thick white setulae plus ten or more shorter dark brown setulae, extended 0.6× distance from upper eye margin to lower eye margin; supracervical setae white; mouthparts geniculated with labella yellow-brown setulose; palpus with brown setulae apically, white setulae on remaining area.

***Thorax*** largely dark brown in ground color with heavy whitish grey pruinosity, generally appearing ash-grey; postpronotal lobe with single dark brown seta, yellow-brown in ground color, therefore, appearing paler than nearby areas; scutum ash-grey with five faint brownish longitudinal bands traceable in clean specimens; two pairs of white scapular setae; acrostical setae widely separated, situated midway between levels of intra-alar setae and postsutural supra-alar setae; post-alar setae same level as intra-alar setae; dorsocentral setae approximately same level as transverse suture; presutural supra-alar setae slightly above level of anterior notopleural setae; two notopleural setae dark brown with posterior seta 0.5× anterior seta; bases of acrostichal, dorsocentral, intra-alar, basal scutellar setae dark brown; scutellum mostly ash-grey but ca. apical 1/5 yellow-brown, slightly convex, almost bare except marginal tiny white setulae; basal scutellar setae more or less parallel, 3.1–3.6× (in males) and 2.4–3.0× (in females) as long as scutellum; apical scutellar setae crossed near apex, 1.1–1.4× (in males) and 0.9–1.1× (in females) as long as scutellum; pleura largely ash-grey; proepisternum with 3–5 white setulae; anepisternum ash-grey with posterior 2/3 white setulose, with single strong dark brown seta and one seta half as long and white ventral to it; katepisternum ash-grey with a strong seta, upper area sparsely covered with short white setulae and lower area with long white setulae; mediotergite ash-grey. Legs yellow-brown ground color with ash-grey pattern and brown to dark brown setae and setulae; fore coxa yellow-brown with posterobasal 1/3 grey, anteriorly with white setulae, posteriorly bare; midcoxa yellow-brown, anteriorly with few long white setulae, posteriorly bare; hind coxa greyish yellow-brown, with white lateral seta, posteriorly largely membranous; femora largely ash-grey except yellow-brown apices; tibiae and tarsi entirely yellow-brown; midtibial spur dark brown, 1.0–1.3× as long as wide.

***Wing*** (Fig. 5A, C, D, F) hyaline with brown to dark brown pattern; area around pterostigma with distinct sexual dimorphism (see next paragraph); cells bc, bm, bcu, alula, anal lobe almost entirely hyaline; cell c mostly hyaline with narrow brown to faint brown medial longitudinal band; cell r_1_ with basal 1/4 hyaline, apical 3/4 dark brown with three large hyaline spots apical to pterostigma; cell r_2+3_ without apical hyaline spot but with two large subapical spots often coalesced, two large hyaline spots posterior to three r_1_ spots, two or three tiny spots apical to them; cell br with basal 2/3 almost hyaline, apically dark brown with 1–3 hyaline spot; cell r_4+5_ with single apical spot and 8–12 variably shaped hyaline spots; cell dm with basal 2/5 almost hyaline, apically dark brown with 4–7 variably shaped hyaline spots; cell m with 5–7 hyaline spots; cell cu_2_ with six or seven large hyaline spots coalesced each other resulting in largely hyaline background with few small brown spots. Wing-thorax ratio 2.4–2.6, subcosta-costa ratio 0.53–0.64, cell r_1_-r_2+3_ ratio 2.2–2.7, cell r_4+5_-r_2+3_ ratio 0.54–0.67. R_4+5_ bare.

***Wing dimorphism.*** Male (Fig. 7A, C) with pterostigma entirely dark brown or at most with tiny hyaline spot; large, more or less elliptic dark brown mid-anterior marking traceable covering pterostigma, cell r_1_ adjacent to pterostigma, basal 1/4 to 3/5 of cell r_2+3_, and anterior areas of cells br and r_4+5_ near crossvein r-m; vein M ratio 0.29–0.43. Female (Fig. 5D, E) with pterostigma dark brown with distinct round hyaline spot; large mid-anterior marking not traceable; such marking interrupted by distinct round hyaline spot on pterostigma and 2–4 small round spots on cell br posterior to it; vein M ratio 0.41–0.53.

**Figure 8.**
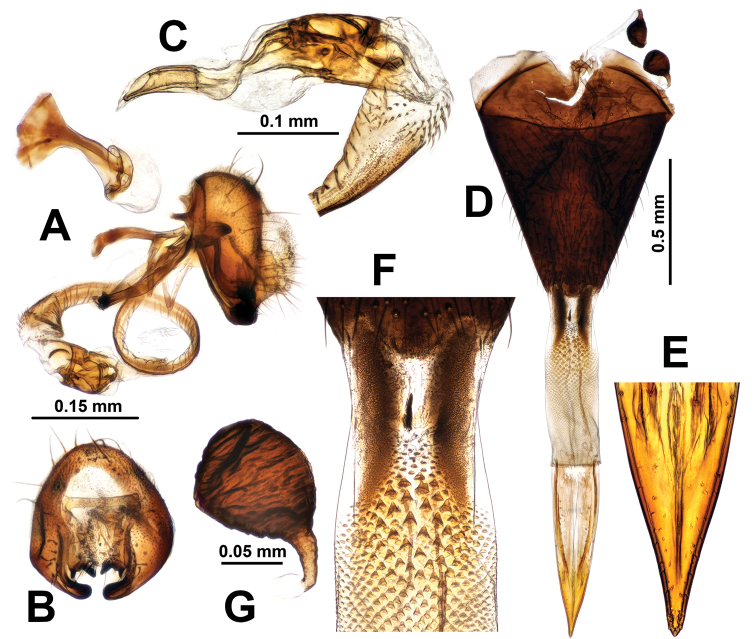
Genitalia of *Campiglossa paramelaena* sp. nov. **A** epandrial complex, lateral view **B** epandrial complex, caudal view **C** glans and preglans of distiphallus **D** female postabdomen with aculeus and eversible membrane pulled out, ventral view **E** magnified view of aculeus tip **F** magnified view of oviscape and eversible membrane **G** spermatheca.

***Male abdomen.*** Preabdomen slightly longer than wide, almost entirely ash-grey; tergites 2–5 with white setulae, but tergite 5 also with 5–7 dark brown marginal setae; tergites 3–5 each with pair of brown submedian spots. Postabdomen (Fig. 8A–C) with proctiger short, 0.4× as long as epandrium in lateral view, microtrichosae, lower half with numerous yellow-brown setae; epandrium plus surstyli oval in caudal view; epandrium dark brown with long yellow-brown to brown setae, microtrichosae; lateral surstylar flange posteriorly serrate, with its basal width approx.1/3 as long as epandrial complex height; medial surstylus with lateral prensiseta approx.2/3 as long as medial prensiseta; preglans area of phallus strongly spinulose; glans without subapical lobe; tube-like acrophallus highly pronounced with apicodorsal opening, approx. half as long as glans; ejaculatory apodeme large, fan-shaped.

***Female abdomen.*** Preabdomen slightly longer than wide, almost entirely ash-grey; tergites 2–6 with white setulae, tergite 6 especially with dark brown marginal setae; tergites 3–6 each with pair of brown submedial spots. Postabdomen (Fig. 8D–G) with shiny dark brown oviscape approx. as long as three preceding tergites; oviscape densely covered by dark brown setulae but without any macrosetae, 1.3× longer than wide, cone shaped, dorsoventrally flattened; eversible membrane with taeniae approx. 1/3 as long as total length of membrane; posterior 2/3 area of eversible membrane densely covered with anteriorly directed triangular spinules; spinules largest in area behind taeniae; aculeus elongated, dorsoventrally flattened, approx. 4× longer than wide with apical 2/5 gradually pointed, apex with pair of tiny subapical teeth; two similar sized dark brown spermathecae, each with pear-shaped apical receptacle with transverse wrinkles and half as long narrow basal neck; spermathecal duct transparent.

##### Distribution.

Korea, the Russian Far East.

##### Remarks.

**Figure 9.**
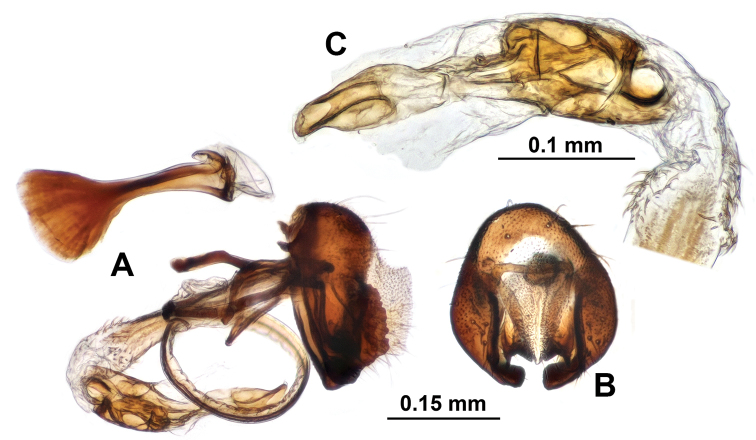
Male genitalia of *Campiglossa melaena***A** epandrial complex, lateral view **B** epandrial complex, caudal view **C** glans and preglans of distiphallus.

Individuals of *C. paramelaena* sp. nov., have DNA barcodes (Figs 1, 2) indistinguishable from those of *C. melaena*, which is a distinctly darker species with a more extensive wing pattern (Fig. 7G–I). Superficially, *C. paramelaena* sp. nov., more closely resembles *C. misella* (see Diagnosis), while the average barcode distance between these two species is 1.9 % (range 1.7–2.1 %). We postulate that *C. paramelaena* sp. nov., is not a light-colored seasonal form of *C. melaena*, because both species are from the same collecting lot in the Russian Far East (see Type material). Moreover, this species not only has a lighter body coloration but also has a much sparser wing pattern on the anal area then in *C. melaena*. In addition, the male surstylar flange of *C. melaena* is relatively larger (the base of the flange is approx. half as long as the height of the epandrial complex in the lateral view) than that of *C. paramelaena* sp. nov. (the base of the flange is distinctly shorter than half the height of the epandrial complex) (Fig. 9A vs. Fig. 8A).

#### Campiglossa melaena

Taxon classificationAnimaliaDipteraTephritidae

(Hering)

51392B10-E9C0-5350-A5C2-8799AB9AEE3A

Figs 5G–K , 9A–C

Sinotephritis melaena Hering, 1941: 27 (Type-locality: China: Manchuria, Sjaolin. Holotype ♂, allotype ♀, NHMUK).
Campiglossa melaena : Korneyev 1990: 443 (new combination); Wang 1998: 255, 265 (in the East Asian Campiglossa key, diagnosis); Norrbom et al., 1999: 112 (in world Tephritidae catalog); Korneyev and Ovchinnikova 2004: 545 (in the Russian Far East Tephritidae key).

##### Material examined

. Russia: Primorsky-Krai: Khasansky-District, Barabash, 43°10'46.9"N, 131°28'20.0"E, 61m, 22.VI.2008, H.Y. Han and H.S. Lee, 3♂ (YSUW); Nadezhdinsky-District, Vol’no-Nadezhdinskoye, grassland near restaurant, 43°22'31.6"N, 132°01'43.1"E, 61m, 22.VI.2008, H.Y. Han and H.S. Lee, 3♂ (YSUW).

##### Diagnosis.

This is the darkest species of the *misella* group, showing the least wing dimorphism (Fig. 7G, I, J vs. K). ***Head*** largely brown with dark grey upper occiput. ***Thorax*** with dark grey scutum with five brownish longitudinal stripes (Fig. 7H); scutellum dark grey; ***Legs*** with coxae and femora largely dark grey but tibiae and tarsi brown; fore femur with 5–7 dark brown posteroventral setae. ***Wing*** almost entirely brown to dark brown with numerous hyaline spots; male with large dark mid-anterior marking covering from pterostigma to posterior end of crossvein R-M; male pterostigma almost completely dark brown, at most with tiny hyaline spot (Fig. 7I-a); female pterostigma with larger hyaline spot (Fig. 7K-a); cell r_1_ posterior to pterostigma with three large hyaline spots in both sexes; cell r_2+3_ with posteroapical hyaline spot (Fig. 7I-b). ***Abdomen*** almost entirely dark grey.

##### Distribution.

North east China, the Russian Far East.

##### Remarks.

Hering’s (1941) original description and wing drawing of the holotype from north east China fall clearly within the variation range of the specimens we obtained from the Russian Far East. Unfortunately, we were not able to collect any female specimens, but Korneyev and Ovchinnikova’s (2004) illustrations (Fig. 7J, K) show a similar sexual dimorphism of the wing pattern as in the other *misella* group species. Individuals of *C. melaena* have DNA barcodes (Figs 1, 2) indistinguishable from those of *C. paramelaena* sp. nov. (see the Remarks of the latter species for further discussion).

### Presumed members of the *misella* group

The following two species are tentatively placed in the *misella* group based only on the superficial male characters available from the original and subsequent descriptions. In the future, their memberships should be confirmed by the female characters as well as a DNA barcoding analysis.

#### Campiglossa pishanica

Taxon classificationAnimaliaDipteraTephritidae

(Wang, 1996)

ABE72D58-B47D-5B5D-8A38-2F490B029997

Fig. 10E

Tephritis pishanica Wang, 1996: 188 (Type-locality: CHINA, Xinjian Province, Pishan, holotype ♂, paratype 2♂, IZAS); Wang 1998: 291, 300 (in the East Asian Tephritis key, diagnosis); Korneyev 2004: 8 (erroneous synonymy with C. misella); Korneyev and Ovchinnikova 2004; 546 (erroneous synonymy with C. misella).

##### Diagnosis.

**Figure 10.**
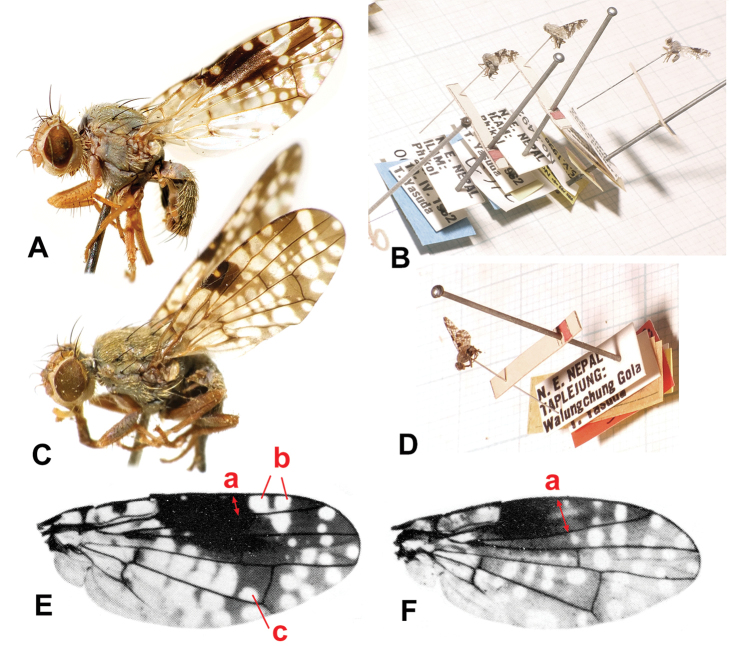
**A** Holotype male of *Campiglossa favillacea* Ito, 2011 (new synonym of *C. coei*, UOPJ) **B** from left, two paratype females and holotype of *C. favillacea* [UOPJ] **C** holotype female of *C. roscida* Ito, 2011 (new synonym of *C. misella*) **D** ditto **E** holotype or paratype male wing of *C. pishanica***F** holotype male wing of *C. propria***E, F** reproduced from Wang (1998) with permission from Xing-Jian Wang.

This is an interesting species showing the characteristics of both *C. coei* and *C. misella*. The only known *C. pishanica* male wing pattern is very similar to that of *C. coei* (Fig. 10E vs. Fig. 4A, C), but Fig. 10E shows the following differences: pterostigma almost completely dark with very tiny hyaline spot (Fig. 10E-a; *C. coei* male consistently has a much larger spot, Fig. 4A-a), and fork of vein Rs and area posterior to it with dark spot. Except for the much lighter basal wing area, *C. pishanica* body appears very similar to that of *C. misella*, which also has dark femora and a scutum with five stripes.

##### Distribution.

Only three males (the type series) known from China (Xinjian).

#### Campiglossa propria

Taxon classificationAnimaliaDipteraTephritidae

(Chen, 1938)

F1E23188-1B0F-54C9-AEC1-0B0DB01AD52B

Fig. 10F

Sinotephritis propria Chen, 1938: 149 (Type-locality: China, s.e. Gansu, Mi-tching-ngai, holotype ♂, IZAS).
Campiglossa propria : Korneyev 1990: 454 (new combination); Wang 1998: 254, 268 (in the East Asian Campiglossa key, diagnosis); Norrbom et al. 1999: 113 (in the world Tephritidae catalog); Korneyev and Ovchinnikova 2004: 544 (in the Russian Far East Tephritidae key).

##### Diagnosis.

We are not sure if this species actually belongs to the *misella* group, because the only known male (holotype) does not show close similarity to any known member of the group except for its large mid-anterior dark wing marking (Fig. 10F). This male also shows an unusual enlargement of cell r_1_ resulting in a distinctly more rounded anterior wing margin than other species (Fig. 10F-a vs. Fig. 10E-a). In addition to this peculiar enlarged cell r_1_, *C. propria* male can also be diagnosed based on the following characteristics: scutum ash-grey with five brownish longitudinal stripes; legs entirely yellowish; cell r_1_ apical to pterostigma with three tiny hyaline spots plus a large subapical hyaline spot; cell r_2+3_ basal to crossvein R-M dark without any spot, apical to R-M with six hyaline spots including posteroapical spot; abdominal tergite 3–5 each with pair of large brown submedian spots.

##### Distribution.

Only known from the type locality (Gansu, China).

## Conclusions

The genus *Campiglossa* currently includes ca. 200 similar looking species with their larvae usually feeding in the capitula of Asteraceae plants (White 1988). Unlike other species-rich pest tephritid genera such as *Bactrocera* Macquart, 1835, and *Anastrepha* Schiner, 1868, most *Camiglossa* species have been considered to be non- or minor economic pests. Therefore, relatively less research effort has been made to investigate *Campiglossa*. Furthermore, their unusually high intraspecific and low interspecific variation (Han 2019) has been a hurdle against establishing a sound classification of the genus *Campiglossa*. We found that the combination of the following taxonomic procedures is useful to investigate this enigmatic genus of the family Tephritidae.

### Collecting and preservation

The male and female flies of most *Campiglossa* species seem to stay close to their host plants (White 1988; pers. obs.), unlike many other lek-mating tephritid taxa whose females only briefly visit their host plants for oviposition (White 1988; Díaz-Fleischer and Aluja 1999; Han 1999). According to our experience, sweep-netting through the area of suspected host plants has been the most productive way of obtaining diverse *Campiglossa* species; the Malaise and lure traps have not been effective methods for collecting this group of flies. Pinned specimens (usually double-mounted) are best for examining external morphological characteristics of *Campiglossa*, because it is difficult to observe the pattern of pruinosity in alcohol-preserved specimens. Postmortem changes can often be a problem as in other tephritids. For example, the brilliant coloration of the eyes disappears quickly in dried or alcohol specimens. In pinned *Campiglossa* specimens, some oily body fluid often oozes out and ruins specimens. In such specimens, observation of the color pattern becomes increasingly difficult. Freeze drying (simply by keeping specimens in a freezer for a few months) can alleviate this problem to some extent, but there seems to be no complete solution.

### Host rearing

Capitula infesting tephritids including *Campiglossa* are the easiest tephritids to rear. Mature flower heads of Asteraceae plants should be collected and kept in mesh bags (similar to insect net bags). These bags should be stored in a sheltered area which maintains an approximate outdoor temperature, and examined for emerging flies. Once the plant materials dry, proper moisture should be maintained by misting with sterile water once or twice a week. In Korea, fall-collected flower heads, after harvesting fall-emerged tephritids, are kept in a 4 °C refrigerator between early December and early April. Overwintered flower heads, if infested by overwintering immature tephritids, usually yield adult flies until early June. Emerged flies should always be kept alive for a few days for hardening and coloring of their cuticles. Each puparium may be separated and kept in a gelatin capsule to match the emerged adult and its own puparium (see White 1988, for more detail). The host-associated *Campiglossa* specimens obtained in this manner have been extremely useful in understanding their inter- and intraspecific variations as well as sexual dimorphism and seasonal variations (Han 2019).

### Photography

**Figure 11.**
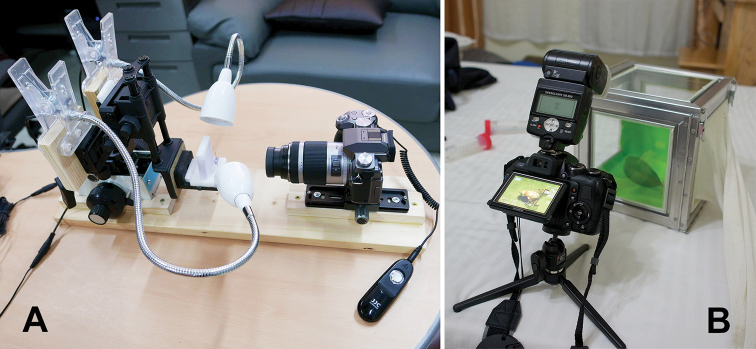
Macro photography setups for multi-day collecting trips. **A** A simple handmade collapsible macro-photography unit for focus stacking **B** A setup for photographing live tephritid flies. Please see the Materials and methods for details of the camera setups.

Due to the postmortem deterioration of the *Campiglossa* specimens, it is desirable to take high resolution photographs while they are still alive or just after euthanasia. Most of the figures presented in this study have been made in this manner. A collapsible glass cage and a simple hand-made macro-photography stacking station are useful for taking such pictures during a multi-day collecting trip (Fig. 11). Male and female terminalia can also be photographed using the focus stacking method.

### DNA barcoding

As demonstrated in this study, DNA barcode sequences of the genus *Campiglossa* form a strong monophyletic clade when analyzed phylogenetically. Therefore, any tephritids of uncertain identity clustering together within this clade should be regarded as *Campiglossa*. For this reason, we synonymize the genera *Dioxyna* and *Homoeotricha*, which were clearly placed within this clade (Figs 1, 2). We also found at least ten major monophyletic lineages within the *Campiglossa* clade and recognize them as ten putative species groups, among which the *misella* group is taxonomically revised here. We postulate that more species groups could be discovered as our DNA barcode dataset increases. Recognizing such a group would be an initial step toward establishing a sound classification of this enigmatic genus of Tephritidae. For the *misella* group, DNA barcoding has also been useful for clarifying their inter- and intraspecific morphological variation, as well as their sexual dimorphism.

## Supplementary Material

XML Treatment for Campiglossa coei

XML Treatment for Campiglossa misella

XML Treatment for Campiglossa paramelaena

XML Treatment for Campiglossa melaena

XML Treatment for Campiglossa pishanica

XML Treatment for Campiglossa propria
